# Impact of Heterogeneity in Sexual Behavior on Effectiveness in Reducing HIV Transmission with Test-and-Treat Strategy

**DOI:** 10.1371/journal.pcbi.1005012

**Published:** 2016-08-01

**Authors:** Ganna Rozhnova, Maarten F. Schim van der Loeff, Janneke C. M. Heijne, Mirjam E. Kretzschmar

**Affiliations:** 1 Julius Center for Health Sciences and Primary Care, University Medical Centre Utrecht, Utrecht, The Netherlands; 2 Department of Infectious Disease Control, Public Health Service Amsterdam, Amsterdam, The Netherlands; 3 Center of Infection and Immunity Amsterdam, Academic Medical Center, Amsterdam, The Netherlands; 4 Centre for Infectious Disease Control, National Institute of Public Health and the Environment, Bilthoven, The Netherlands; Duke University, UNITED STATES

## Abstract

The WHO’s early-release guideline for antiretroviral treatment (ART) of HIV infection based on a recent trial conducted in 34 countries recommends starting treatment immediately upon an HIV diagnosis. Therefore, the test-and-treat strategy may become more widely used in an effort to scale up HIV treatment and curb further transmission. Here we examine behavioural determinants of HIV transmission and how heterogeneity in sexual behaviour influences the outcomes of this strategy. Using a deterministic model, we perform a systematic investigation into the effects of various mixing patterns in a population of men who have sex with men (MSM), stratified by partner change rates, on the elimination threshold and endemic HIV prevalence. We find that both the level of overdispersion in the distribution of the number of sexual partners and mixing between population subgroups have a large influence on endemic prevalence before introduction of ART and on possible long term effectiveness of ART. Increasing heterogeneity in risk behavior may lead to lower endemic prevalence levels, but requires higher coverage levels of ART for elimination. Elimination is only feasible for populations with a rather low degree of assortativeness of mixing and requires treatment coverage of almost 80% if rates of testing and treatment uptake by all population subgroups are equal. In this case, for fully assortative mixing and 80% coverage endemic prevalence is reduced by 57%. In the presence of heterogeneity in ART uptake, elimination is easier to achieve when the subpopulation with highest risk behavior is tested and treated more often than the rest of the population, and vice versa when it is less. The developed framework can be used to extract information on behavioral heterogeneity from existing data which is otherwise hard to determine from population surveys.

## Author Summary

HIV is endemic in populations of MSM in Western countries. As ART reduces transmission risk, increased testing and treatment rates are expected to lower HIV incidence. However, concerns are that in MSM populations changing risk behavior may counteract the impact of ART on transmission. Using a mathematical model, we investigated how heterogeneity in sexual behavior influences the possible effects of a test-and-treat strategy on HIV prevalence and in particular the prospects of eliminating HIV from these populations. We demonstrated that behavioral heterogeneity plays an important role in determining the impact of ART on reducing HIV transmission. Knowledge of behavioral heterogeneity is key in setting intervention goals in populations of MSM.

## Introduction

Recently, a large trial conducted at various sites in 34 countries provided evidence that starting ART as soon as possible regardless of CD4 count is advantageous for health prospects of HIV infected persons [1]. The WHO’s early-release guideline for ART initiation now reflects these findings recommending to start treatment immediately upon an HIV diagnosis [2]. Therefore, the test-and-treat strategy, where a population is tested for HIV regularly and those found positive are treated immediately, may become widely used in countries with a generalized HIV epidemic. Earlier, it was investigated whether and under which circumstances a test-and-treat strategy and a more general strategy of treatment as prevention would eventually lead to elimination of HIV from a population [3–8]. While much discussion has been devoted to the possible influence of high infectiousness during primary HIV infection on expected effects of large scale ART on HIV incidence [9–12], less attention has been directed to behavioural determinants of HIV transmission dynamics and how heterogeneity in sexual behaviour will influence the impact of a test-and-treat strategy on HIV transmission. Models of HIV treatment as prevention already included heterogeneity (e.g. [7]) but there has been no systematic investigation of how results depended on assumptions about it.

Sexual behaviour influences HIV transmission dynamics in various ways. Changes in sexual risk behaviour over time have been observed, first decreasing risk behaviour as a response to the emerging HIV epidemic in the 1980’s, and later increasing risk when ART became available at the end of the 1990’s. These changes have been especially apparent in populations of MSM [13–16]. More recently, modelling studies showed the impact of changes in risk behaviour of individuals over time on HIV transmission dynamics [17–27].

Here we developed a modeling approach which allows a systematic investigation into the effects of various mixing patterns in populations stratified by rates of partner change on the basic reproduction number, treatment effects and prospects of elimination. We investigated how endemic levels and elimination threshold depend on the level of overdispersion in the distribution of numbers of partners and on a mixing parameter that reflects assortativeness of mixing. We studied how the infection is distributed across population strata in endemic steady state and how this changes with various levels of diagnosis and treatment. We chose baseline parameter values to reflect the HIV epidemic among MSM in Western countries.

## Materials and Methods

### Model formulation

We considered an extended version of a previous model used to evaluate prospects of elimination of HIV with test-and-treat strategy [5]. Here, we explicitly incorporated risk heterogeneity in sexual activity and mixing between population groups by sexual activity.

The model represents a population of MSM of size *N*(*t*) stratified into *m* risk groups of size *N*_*l*_(*t*) with partner change rates *c*_*l*_, *l* = 1, …, *m*. Here $N\left(t\right)=\sum_{l=1}^{m}N_{l}\left(t\right)$ is the time-dependent population size that changes due to additional mortality from HIV infection. Individuals remain in their risk group. The population in group *l* consists of susceptible, *S*_*l*_(*t*), infected, *I*_*lk*_(*t*), and treated, *A*_*lk*_(*t*), individuals in stage of infection *k* = 1, …, *n*, where *n* is the number of disease stages. The population size in group *l* can then be expressed as $N_{l}\left(t\right)=S_{l}\left(t\right)+\sum_{k=1}^{n}[I_{lk}\left(t\right)+A_{lk}\left(t\right)]$.

**Fig 1:**
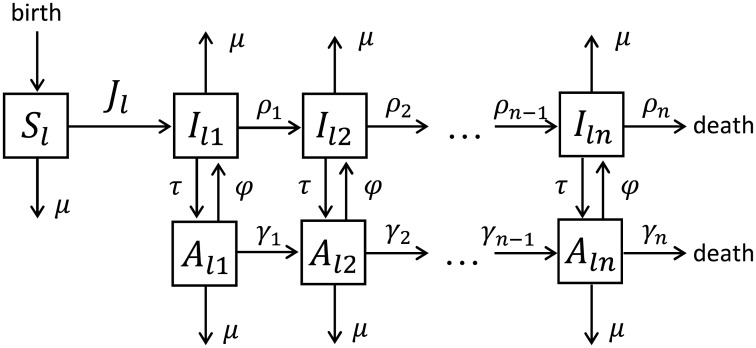
Schematic diagram of the HIV model. The model describes HIV infection process, disease progression through *n* stages of infection, birth, background mortality and HIV related mortality, the uptake of and dropping out of ART. The model assumes that the population is divided into the classes of susceptibles, *S*_*l*_(*t*), infected, *I*_*lk*_(*t*), and treated, *A*_*lk*_(*t*), in *n* stages of infection, *k* = 1, …, *n*. The population is stratified into *m* risk groups indicated by the label *l* = 1, …, *m* in the subscript, and the diagram describes the dynamics in one of them. The risk groups differ only in their partner change rates and initial group sizes, whilst the disease progression and dropping out of treatment are the same for all risk groups. The diagram shows the situation when treatment uptake is the same by all risk groups. We denote *τ*_*l*_ the uptake by risk group *l* when we consider heterogeneous ART uptake by risk group. The interaction between the groups is encoded in the time-dependent force of infection *J*_*l*_(*t*) that takes into account mixing between the risk groups.

In each risk group the model describes HIV infection process, disease progression through *n* stages of infection, birth, background and HIV related mortalities, the uptake of and dropping out of ART (Fig 1). Individuals enter the risk class *l* at rate $\mu N_{l}^{0}$ as susceptible, where $N_{l}^{0}$ is the initial number of individuals in group *l*. Susceptibles can become infected with the first stage of HIV infection at rate *J*_*l*_(*t*) (force of infection). In the absence of treatment infected individuals progress through *n* stages of infection with varying durations and infectivities ending with death from HIV. Infected individuals in any stage can be screened and start ART at rate *τ*. When we will consider heterogeneous ART uptake by risk group, we will denote *τ*_*l*_ the uptake by risk group *l*. Treated individuals progress through *n* stages of infection with varying durations too, albeit at smaller rates. The rates of progression from stage *k* to stage *k* + 1 for untreated and treated individuals are denoted as *ρ*_*k*_ and *γ*_*k*_, respectively. Treated individuals in stage *k* revert to an infection of stage *k* at rate *ϕ*. This transition represents leaving the virally suppressed state; this can be due to treatment failure, dropping out of treatment or other reasons. In the following, we will refer to *ϕ* as drop out rate. Finally, all classes of individuals are subject to background mortality at rate *μ*.

The model was formulated as a system of differential equations for the number of individuals in different classes as follows

$$\frac{dS_{l}\left(t\right)}{dt}=\mu N_{l}^{0}-\mu S_{l}\left(t\right)-J_{l}\left(t\right)S_{l}\left(t\right),$$ (1)

$$\frac{dI_{l1}\left(t\right)}{dt}=J_{l}\left(t\right)S_{l}\left(t\right)-\left(\mu +\rho_{1}+\tau \right)I_{l1}\left(t\right)+\phi A_{l1}\left(t\right),$$ (2)

$$\frac{dI_{lk}\left(t\right)}{dt}=\rho_{k-1}I_{l,k-1}\left(t\right)-\left(\mu +\rho_{k}+\tau \right)I_{lk}\left(t\right)+\phi A_{lk}\left(t\right),$$ (3)

$$\frac{dA_{l1}\left(t\right)}{dt}=\tau I_{l1}\left(t\right)-\left(\mu +\gamma_{1}+\phi \right)A_{l1}\left(t\right),$$ (4)

$$\frac{dA_{lk}\left(t\right)}{dt}=\tau I_{lk}\left(t\right)+\gamma_{k-1}A_{l,k-1}\left(t\right)-\left(\mu +\gamma_{k}+\phi \right)A_{lk}\left(t\right),$$ (5)

where *k* = 2, …, *n* and *l* = 1, …, *m*. For heterogeneous uptake by risk group *τ* has be substituted by *τ*_*l*_ in Eqs (1)–(5).

The time-dependent force of infection (per year) in risk group *l* is given by

$$J_{l}\left(t\right)=\lambda c_{l}\sum_{l^{\prime }=1}^{m}M_{ll^{\prime }}\left(t\right)\sum_{k=1}^{n}\left[h_{k}\frac{I_{l^{\prime }k}\left(t\right)}{N_{l^{\prime }}\left(t\right)}+\epsilon \frac{A_{l^{\prime }k}\left(t\right)}{N_{l^{\prime }}\left(t\right)}\right].$$

Here *λ* is the transmission probability per partnership, *ϵ* is the infectivity for an individual on ART, whereas *h*_*k*_ describe the infectivity in stage *k* of infection. Infectivity is defined as a dimensionless quantity describing the relative contribution of each disease or treatment stage to overall infectiousness.

The *m* × *m* mixing matrix **M**(*t*) = [*M*_*ll*′_(*t*)]_*l*,*l*′ ∈ {1,…,*m*}_ with the elements *M*_*ll*′_(*t*) denoting mixing of susceptible in the risk group *l* with infected in risk group *l*′ is defined as follows

$$M_{ll^{\prime }}\left(t\right)=\omega \frac{c_{l^{\prime }}N_{l^{\prime }}\left(t\right)}{\sum_{l^{\prime \prime }=1}^{m}c_{l^{\prime \prime }}N_{l^{\prime \prime }}\left(t\right)}+\left(1-\omega \right)\delta_{ll^{\prime }},$$ (6)

where *δ*_*ll*′_ = 1 if *l* = *l*′ and *δ*_*ll*′_ = 0 otherwise.

The mixing parameter 0 ≤ *ω* ≤ 1 describes the degree of assortative mixing by risk level. When *ω* = 0 mixing between the risk groups is fully assortative (like with like), when *ω* = 1 mixing is fully proportionate. Eq (6) means that a proportion (1 − *ω*) of the partnerships are formed only with the individuals of the same risk group *l* = *l*′, whereas the remaining proportion *ω* of the partnerships is formed with each risk group (*l*′ = 1, …, *m*) proportionally to the number of partnerships offered by those risk groups. Mixing between groups was random meaning that we did not incorporate preferential mixing for adjacent risk groups. This method of incorporating mixing between different population subgroups is commonly used in sexually transmitted infections (STI) models [11, 28–31].

The proportion of new susceptible individuals entering each risk group was chosen such that, in the absence of HIV, this proportion would remain constant. In the model the total population size, *N*(*t*), however, as well as the population sizes of *m* risk groups, *N*_*l*_(*t*), *l* = 1, …, *m*, are not constant because of additional mortality from HIV infection. Note that the burden of HIV due to HIV related mortality is different per risk group as the groups with the highest number of infected individuals will have more HIV related deaths. S1 and S2 Figs show the time-dependent behavior of the model variables for the default parameter values without and with ART, respectively.

The mathematical model was implemented in *Mathematica 9.0*.

### Model parametrization

For parameterizing the model we chose values that are plausible for describing populations of MSM in Western countries, but it was not our aim to fit the model to a specific population. We used data to choose the order of magnitude for parameters. Estimates for parameter values relating to heterogeneity in sexual behavior were obtained from Rutgers World Population Foundation (WPF) data on MSM collected in 2005–2006 in the Netherlands and previous studies of STI dynamics among MSM in the UK. Parameters relating to disease progression and infectivity were extracted from published literature. The parameters for the model and their baseline values are summarized in S1 Table.

We assumed that the rate of recruitment to the sexually active population, *μ*, equals the death rate. The average duration of sexual activity is 1/*μ* = 45 years [32–34]. We made use of Rutgers WPF data on the number of MSM in the Netherlands as the baseline value for the total population size in the beginning of HIV epidemic, *N*_0_ [35, 36]. The model can accommodate any number of risk groups, *m*. Here we focused on the case *m* = 6 considered previously in modeling dynamics of Hepatitis B virus in MSM populations in the UK and the Netherlands [33, 34, 36–38]. From these studies we adopted the initial fractions of the population in the 6 risk groups, *q*_*l*_, where *q*_*l*_ ≤ 1 for *l* = 1, …, 6 and $\sum_{l=1}^{6}q_{l}=1$. We calculated the initial numbers of individuals in each risk group, $N_{l}^{0}$, from the relation $N_{l}^{0}=q_{l}N_{0}$.

Any number of HIV stages, *n*, can be incorporated into the model. Following Refs. [3, 5, 39] we parametrized the model for the case *n* = 4, because for this choice of *n* estimates for the rates of transition between infection stages for untreated, *ρ*_*k*_, and treated, *γ*_*k*_, individuals, as well as for the infectivities of untreated, *h*_*k*_ (all parameters for *k* = 1, 2, 3, 4), and treated, *ϵ*, individuals were available. It should be noted that these infectivities were estimated for heterosexual couples but we used them for MSM in the absence of similar estimates for this population. For *n* = 4, infection stages are primary infection, asymptomatic chronic stage, the last two stages together define the symptomatic AIDS stage which is subdivided into an infectious and a noninfectious period (due to severe illness leading to cessation of sexual activity). In the model the stages of the population under treatment have no biological interpretation. They were chosen in our previous work (Ref. [5]) such that the survival probability has a distribution function that agrees with CASCADE data from the time period after introduction of ART.

The rate of treatment uptake, *τ*, and the rate of dropping out from treatment, *ϕ*, can be varied in the model. We present the results in terms of annual treatment uptake and dropout percentages, τ* and ϕ*, respectively. These were computed from the expression for the probability that an event (treatment or dropping out) takes place within one year as *percentage* = (1 − e^−*rate*×1 year^)100%. In case of heterogeneous uptake by risk group, the uptake percentage by group *l*, $\tau_{l}^{*}$, is computed using the same expression. Unless stated otherwise, the annual dropout percentage was fixed throughout the analysis at 5%.

The mixing parameter quantifying the degree of assortativeness, *ω*, is a variable parameter of the model. Data on the mixing between different risk groups are hard to obtain because information about the characteristics of sexual partners is required. In STI modeling studies, including HIV, the value of *ω* has been either assumed to have a certain value [29, 30] or estimated by fitting a model to incidence data using Bayesian analysis [11, 31]. The direct estimates of mixing were obtained in three studies of sexual behavior based on contract tracing among patients of STI clinics in the USA and in three studies based on a survey of the general population in the USA, France and the UK [40–42]. These estimates indicate weak like-with-like mixing. However, they do not generalize to the USA MSM population, and, to our knowledge, no more data on mixing is available for this population in the USA or other countries. In our analysis, *ω* is a free parameter that takes on the whole range of possible values, *ω* ∈ [0, 1].

We fixed *λ* at 5% such that HIV prevalence [43] and *R*_0_ [5] in our study represent a plausible range of values that is compatible with HIV dynamics in MSM in Western countries.

### Estimation of partner change rates

To estimate partner change rates, *c*_*l*_, *l* = 1, …, 6, we used Rutgers WPF sexual behavior data for MSM population in the Netherlands [35]. In the Rutgers WPF survey, respondents were recruited via an existing internet panel. Since there were few MSM in the panel, MSM were additionally recruited via banners on websites that are frequently visited by MSM. This resulted in the final list of respondents. Numbers of respondents with certain demographic characteristics (sex, age, education, residence) were matched with the distribution in the population of the Netherlands, such that the survey population was representative in these demographic variables. Younger age groups (15–45 years) and certain ethnic minorities were oversampled. Therefore weighting factors were then used by Rutgers WPF to come to a representative sample with respect to the above variables. From this survey population, we extracted male respondents who reported sex with men. We used data on the self-reported number of partners in the last 6 months including information about a steady partner in the last 6 months and condom use for 176 respondents aged 15 to 70 years. The question respondents answered was: “How often did you use condoms in the last 6 months with casual partners with whom you had anal sex?” A similar question was posed for steady partners. As we estimated the number of new partners, the number of partners was corrected (minus 1) if a person reported a steady partner, and the duration of relationship with this partner was longer than 6 months. Condom use was encoded in a binary variable (0 = always, 1 = not always), where all respondents who reported condomless sex in the last 6 months were grouped into one category. This variable was used as a multiplication factor for the number of partners, so that individuals who always used condoms effectively had 0 partners.

**Fig 2:**
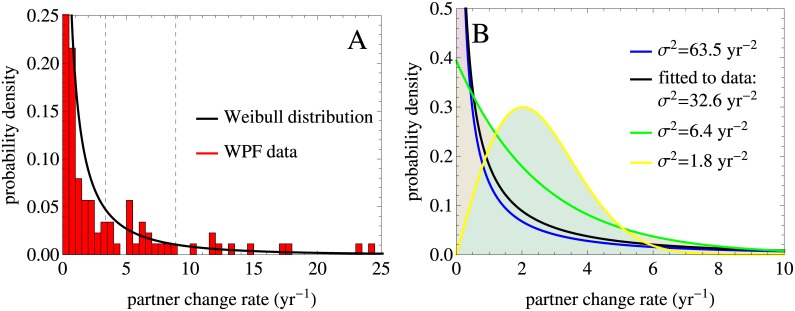
Distributions in the partner change rate used in the analysis have the same mean rate estimated from the data and different variances. (A) Probability density function for the Weibull distribution in the partner change rate fitted to the WPF data histogram by maximum likelihood method. The dashed lines indicate the intervals defined by the initial fractions of the population in the 6 risk groups, *q*_*l*_, *l* = 1, …, 6, per which mean rates of partner change were estimated. For a better visualization the ranges of the x and y axes differ among the panel A and the panel B that is why not all dashed lines can be seen, see S3 Fig for more detail. (B) Weibull distributions with the same mean of *c* = 2.54 partners per year and different variances, *σ*^2^, were obtained by varying the shape parameter, *α*, and the scale parameter, *β*. The Weibull distribution that best fits to the data is shown in black.

We fitted the probability density function for a Weibull distribution to the WPF data histogram by maximum likelihood method. The resulting Weibull distribution is a continuous probability distribution with two parameters, a shape parameter *α* = 0.5 and a scale parameter *β* = 1.26, both of which were obtained from the fitting procedure. Fig 2A shows the probability density function and S3 Fig shows the corresponding cumulative distribution function. From this distribution we obtained the mean rate of partner change, *c* = 2.54 partners per year, and mean rates of partner change, *c*_*l*_, per intervals defined by the initial fractions of the population in the 6 risk groups, *q*_*l*_, *l* = 1, …, 6. Fitting a Gamma distribution with two parameters resulted in similar estimates of the mean partner change rate and of the mean rates in different risk groups, see S4 Fig for details.

Following standard theory, the basic reproduction number for the SIR model with demography and constant population size in a population stratified by partner change rates and with proportionate mixing is proportional to *σ*^2^/*c*, where *σ*^2^ and *c* are the variance and the mean of the distribution in the partner change rate [44]. To study the impact of heterogeneity in partner change rates on the dynamics of the model, we fixed the mean rate and varied *α* and *β* to obtain Weibull distributions with different variances, *σ*^2^. The mean rates of partner change for each of the distributions, *c*_*l*_, were then computed as before (S2 Table). The variance of the distributions in our analysis ranged from 1.8 to 63.5 yr^−2^, see Fig 2B for probability density functions and S3 Fig for the corresponding cumulative distribution functions. The estimate of the variance obtained from the Weibull distribution best-fitting to the data was *σ*^2^ = 32.6 yr^−2^.

### Computation of the effective reproduction number

We analyzed the threshold behavior of the model using the next-generation matrix approach. An extensive discussion of this approach has already been given at length in the literature in the context of compartmental epidemic models, and we refer the reader to [45, 46] for formal details. In S1 Text we described the aspects that are important for this paper. In essence, the method allowed us to compute a parameter, known as the basic reproduction number, *R*_0_, from Eqs (1)–(5). Following standard theory [44], if *R*_0_ > 1 the infection will reach an endemic equilibrium as *t* → ∞, whereas if *R*_0_ < 1 the infection cannot spread in a population. Therefore, *R*_0_ is a threshold parameter for the model. We distinguish *R*_0_ from *R*_*e*_: the effective reproduction number in a population with treatment. Here we use the term “effective reproduction number” to determine the threshold below which HIV cannot persist in a population under treatment. This is different from the reproduction number in the transient phase of the epidemic. The latter is influenced by density dependent effects, the former is not. Before ART was introduced, the HIV epidemic in populations with persistent HIV transmission was characterized by *R*_0_ > 1. As we will see, treatment lowers *R*_0_ and thus *R*_*e*_ ≤ *R*_0_. Elimination by treatment occurs if *R*_*e*_ < 1. The computation of both quantities is similar, but in terms of interpretation it is more clear if we distinguish these two, see S1 Text for the details.

### Lorenz curves

To describe the distribution of infections across risk groups for populations with different levels of heterogeneity and mixing we used a method based on the so-called Lorenz curve. This method was shown to be useful for calibrating STI models that include risk structure [47]. The Lorenz curve is a graphical representation of a cumulative probability distribution, namely it represents the cumulative proportion of HIV infected individuals as a function of the cumulative proportion of the population in different risk groups ranked in the order of their partner change rate. In the model the population sizes of different risk groups change [over time] as a result of the differential burden of HIV in each risk group, so in principle we could use the cumulative proportion either of the initial or of the final population as the x-axis. We checked that Lorenz curves were not affected much by this choice, therefore we used the initial population fractions in our analysis (S1 Table). The skewness in the distribution of HIV infections across the risk groups is measured by the deviation of the Lorenz curve from the diagonal line. The diagonal denotes the symmetric situation, i. e. the situation where every risk group has the same HIV prevalence.

## Results

We present results regarding the elimination threshold, as given by the effective reproduction number *R*_*e*_, the endemic prevalence and the Lorenz curves. First, we analyzed the model without treatment for a range of distributions in the partner change rate with the same mean rate and different variances. Then, we introduced ART (and consequently dropping out from ART too) into the model and focused on the distribution of partner change rate fitted to the Rutgers WPF data. The prevalence was computed in the steady state. In the case when there is treatment, prevalence is computed as the percentage of all infected individuals including those on ART.

### Analysis without ART

**Fig 3:**
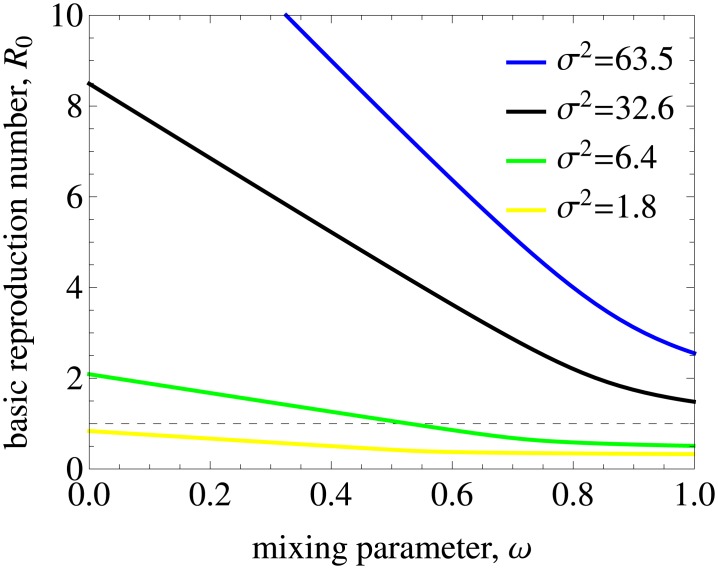
The impact of mixing on the basic reproduction number in the model without treatment. The results are for populations with different variances in partner change rates, *σ*^2^, and the mean rate of partner change kept constant. Mixing is proportionate (assortative) for *ω* = 1 (*ω* = 0). The dashed line indicates the threshold value of *R*_0_ = 1 below which HIV cannot spread in the population. *R*_0_ decreases as the mixing becomes proportionate and the variance gets lower. For low variances and high levels of mixing (i.e. low levels of assortativeness) *R*_0_ can be smaller than 1 even in the absence of treatment. For the lowest variance we used in the analysis HIV cannot persist for any level of mixing (the yellow curve).

Fig 3 shows the basic reproduction number *R*_0_ as a function of the mixing (assortativeness) parameter *ω* in the model without treatment. We observe that *R*_0_ has a strong dependence both on *ω* and on the heterogeneity of the population as quantified by the variance in the rate of partner change, *σ*^2^. Our model predicts that *R*_0_ is below 1 for populations with a low variance and low levels of assortativeness even in the absence of treatment. For the lowest variance used in the analysis, *σ*^2^ of 1.8 yr^−2^, HIV cannot persist in the population for any level of mixing.

**Fig 4:**
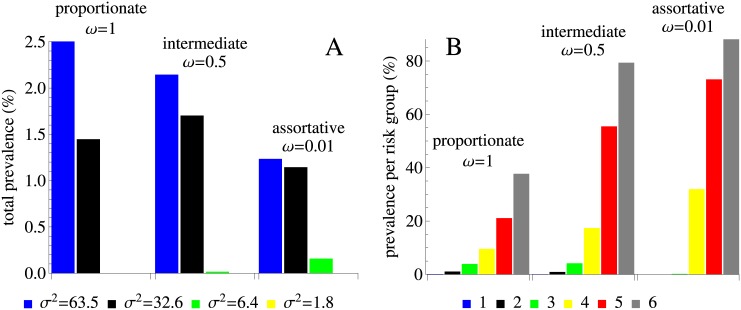
The impact of mixing on endemic prevalence in the model without treatment. (A) Total prevalence in the population. The color of the bars corresponds to the variance in the rate of partner change, *σ*^2^. Note that prevalence is zero for the parameters where HIV is not able to spread (yellow curves for all levels of mixing and the green curve for proportionate mixing in Fig 3). (B) Prevalence per risk group for a population with a variance in the rate of partner change *σ*^2^ of 32.6 yr^−2^ corresponding to the data. The color of the bars denotes the number of the sexual activity class.

Fig 4 demonstrates the impact of mixing on prevalence. For a fixed variance, prevalence does not necessarily change monotonically as mixing ranges from proportionate to intermediate to assortative (*ω* ranges from 1 to 0), see Fig 4A. For example, for *σ*^2^ = 63.5 yr^−2^, the total prevalence is highest for proportionate mixing (blue bars) but for a lower variance, *σ*^2^ = 32.6 yr^−2^, prevalence is highest for intermediate levels of mixing (black bars). This nontrivial effect occurs because population sizes of subgroups are not constant due to HIV related mortality (see S5 Fig). For populations with high variance, prevalence is reduced by more than half (from 2.5% to 1.2%) as the mixing changes from proportionate to assortative. The assortativeness parameter quantifies the extent to which different risk groups of the population are coupled. As *ω* decreases HIV is able to persist in a lower number of risk groups but prevalence per risk group gradually gets higher (Fig 4B). The increase in prevalence with an increasing partner change rate is not unexpected and corroborates the concept of a ‘core’ group.

**Fig 5:**
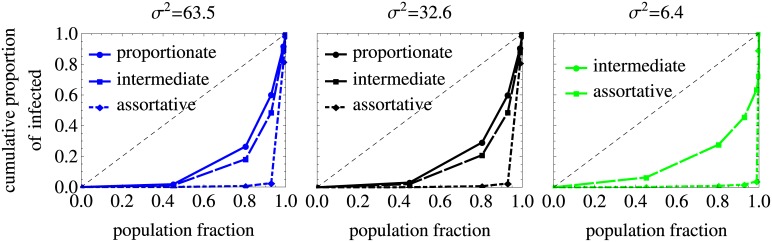
Lorenz curves in the model without treatment. The results are for populations with different variances in partner change rates, *σ*^2^, and the mean rate of partner change kept constant. The diagonal line represents the situation in which every risk group would have the same HIV prevalence. Lorenz curves deviate from it which means that the distributions of infection across the risk groups for proportionate, intermediate and assortative mixing are skewed with high prevalence in small high risk groups. This effect gets stronger as the mixing becomes more assortative. Note that we did not plot the results for the parameters used in Fig 3 for which HIV is not able to spread (yellow curves for all levels of mixing and the green curve for proportionate mixing).

In Fig 5 we plot the Lorenz curves that represent the cumulative proportion of infected individuals as a function of the cumulative proportion of the initial population when the risk groups are ranked in the order of their average number of partners per year. The diagonal line represents the situation in which every risk group would have the same HIV prevalence. The Lorenz curves deviate significantly from the diagonal, indicating that the infection is concentrated in the groups with the highest numbers of partners. This skewness in the distribution of HIV is more pronounced for higher assortativeness of mixing as seen from the comparison of the solid, dashed and dot-dashed curves in the plot.

### Impact of ART on HIV dynamics

**Fig 6:**
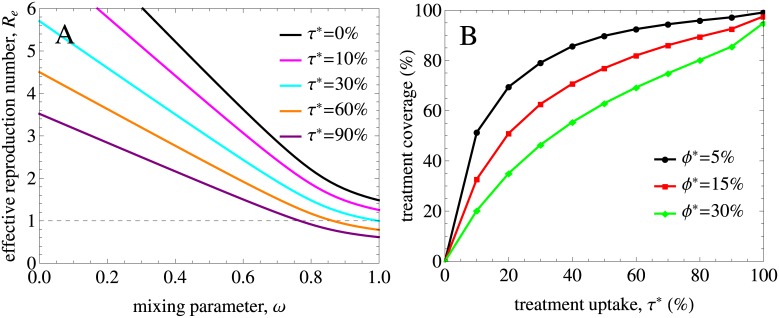
Effective reproduction number and treatment coverage for different annual treatment uptakes and dropout percentages. (A) The impact of annual treatment uptake, τ*, on the effective reproduction number for a population with the variance in the rate of partner change corresponding to the data. Mixing is proportionate (assortative) for *ω* = 1 (*ω* = 0). The curve plotted for τ* = 0.0 is, by definition, *R*_0_. The dashed line indicates the threshold value of *R*_*e*_ = 1 below which HIV is eliminated from the population. *R*_*e*_ decreases with increasing treatment uptake and mixing. (B) Treatment coverage as a function of annual treatment uptake τ* for different dropout percentages ϕ* and the remaining parameters as in A.

Treatment is able to decrease the basic reproduction number and eliminate HIV if *R*_*e*_ gets below 1. In Fig 6A *R*_*e*_ is shown as a function of annual treatment uptake τ* and mixing. The model predicts that for proportionate mixing elimination is feasible in populations with an annual treatment uptake above 30%. The range of *ω* where *R*_*e*_ < 1 gets wider with increasing τ*. Nonetheless, it is not feasible to eliminate HIV from populations with high degree of assortativeness without additional intervention measures even if treatment uptake is as high as 90% annually if treatment uptake is the same in all risk groups.

To translate these findings into results on treatment coverage in the population required for HIV elimination, we show in Fig 6B the coverage as a function of annual treatment uptake for different dropout percentages. Here, the coverage is defined as percentage of infected individuals who are on treatment in the steady state, —a measure that can be obtained from HIV data on diagnosis and treatment. Note that in our model the infected population includes those who are unaware of their infection. Our results indicate that annual treatment uptake of more than 30% required for elimination corresponds to a coverage of almost 80% if 5% drop out from ART due to treatment failure or other reasons annually. For mixing with a higher degree of assortativeness, treatment coverage has to be even higher. This 80% coverage is in line with the UNAIDS 90/90/90 treatment target according to which 90% of all people living with HIV will know their HIV status, 90% of all people with diagnosed HIV infection will receive sustained ART and 90% of all people receiving ART will have viral suppression by 2020 [48]. The first two objectives lead to a coverage of 90% × 90% = 81% meaning that it may be possible to reach elimination for a realization of these objectives in relatively homogeneous populations, but not in populations with strong heterogeneity in sexual behavior and mixing.

**Fig 7:**
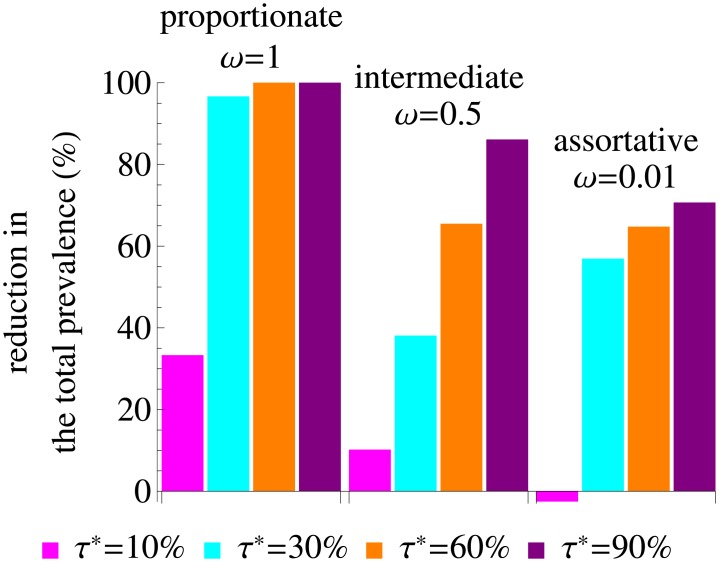
Percentage reduction in the total prevalence due to treatment. The variance in the rate of partner change equals that of the data. The color of the bars corresponds to the annual treatment uptake, τ*.

In Fig 7 we show the percentage reduction in the total HIV prevalence due to treatment. The reduction is 100% for proportionate mixing and annual ART uptake above 60%, meaning that for these parameters we can achieve elimination. For other types of mixing patterns elimination is not feasible but the reduction in prevalence is still significant if treatment uptake is sufficiently high. The reduction is 38% and 57% for τ* = 30% and intermediate and assortative mixing, respectively, and even higher for higher values of τ*. In some cases, however, treatment can have even a slight adverse effect on prevalence, as for τ* = 10% and assortative mixing. This happens because this treatment uptake is not sufficient to decrease HIV transmission substantially when different risk groups do not interact whilst the average lifespan of individuals on ART, and thus the total number of infected individuals, increase. In the model with treatment we again find skewness in the distribution of infections among risk groups that gets more pronounced with decreasing *ω*, see Lorenz curves in S6 Fig. ART uptake has only modest impact on this distribution, with its shape almost entirely defined by the type of mixing pattern.

### Heterogeneous uptake of testing and treatment

**Fig 8:**
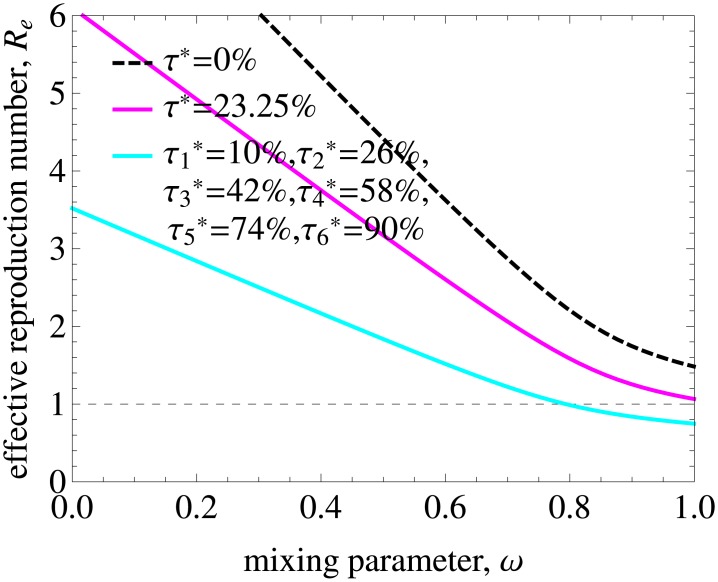
Effective reproduction number for heterogeneous uptake of testing and treatment. $\tau_{l}^{*}$ denotes the uptake by risk group *l*. We considered progressively higher uptake rates by groups with higher numbers of partners. Specifically, we assumed that $\tau_{1}^{*}=10\%$ (lowest risk group), $\tau_{6}^{*}=90\%$ (highest risk group), and uptakes by the remaining 4 groups were equally spaced and increasing from 26% to 74%. The dashed line is *R*_0_ before ART. Also shown is *R*_*e*_ for a constant treatment uptake rate of 23.25% computed as an average of uptakes by different groups weighted by their population size, $\sum_{l=1}^{6}q_{l}\tau_{l}^{*}$. Heterogeneous test-and-treat with increasing treatment uptakes by higher risk groups has a larger impact on *R*_*e*_ than homogeneous test-and-treat with a constant average uptake. See also S7 Fig with results for other combinations of treatment uptakes by different risk groups.

The national survey data on HIV testing and risk behavior in Britain shows that voluntary confidential HIV testing by men is significantly associated with reporting greater numbers of same-sex partners [50]. We thus investigated how heterogeneity in uptake of testing and treatment possibly affect our conclusions regarding the levels of ART necessary for elimination. In Fig 8 we considered progressively higher uptake rates by groups with higher numbers of partners. Specifically, we assumed that uptakes by the lowest and highest risk groups, $\tau_{1}^{*}$ and $\tau_{6}^{*}$, were 10% and 90%, respectively, and uptakes by the remaining 4 groups were equally spaced and increasing from 26% to 74% (indicated in the figure legends). In this case, elimination was possible for populations with values of mixing parameter above 0.8, i. e. for populations with mixing closer to proportionate. Heterogeneous testing and treatment offers much better prospects for HIV elimination than a constant treatment uptake rate of 23.25% computed as an average of uptakes by different groups weighted by their population size, $\tau^{*}=\sum_{l=1}^{6}q_{l}\tau_{l}^{*}$ (Fig 8). For this level of uptake elimination was not feasible at all. However, very high treatment uptakes in the highest risk groups amount to even higher treatment coverages in those groups, which would require intense screening programmes. The results for other combinations of treatment uptakes by different risk groups are shown in S7 Fig. There we show that *R*_*e*_ has values above 1 (elimination is unfeasible) in a wider range of mixing parameter when ART uptake by highest risk individuals is smaller than by the rest of the population, and vice versa if they are tested and get treated more frequently.

### Sensitivity analysis

#### Ratio of primary to chronic infectivity

**Fig 9:**
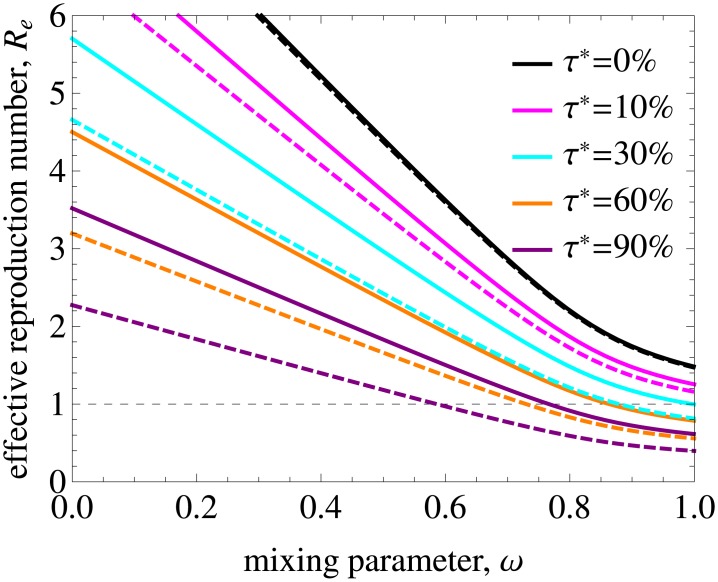
Effective reproduction number as a function of mixing and treatment uptake for low and high ratios of primary to chronic infectivity. The solid lines are repeated from Fig 6A and correspond to the baseline value of the ratio *h*_1_/*h*_2_ = 26.04. The dashed lines were obtained for *h*_1_/*h*_2_ = 4.65 by shifting infectivity from primary to chronic infection while retaining a constant total infectivity. Elimination can be achieved for smaller values of treatment uptake percentage and in a wider range of mixing parameter if the infectivity of the primary phase is lower.

The analysis presented so far implied that the ratio of primary to chronic infectivity, *h*_1_/*h*_2_, is according to estimates obtained by Hollingsworth et al [39], namely the primary phase is about 26 times as infectious as the chronic stage (*h*_1_ = 2.76 and *h*_2_ = 0.106, S1 Table). A recent study indicates that this ratio may be as low as 5 (*h*_1_ = 0.62 and *h*_2_ = 0.12, [49]) and that the duration of primary phase is 1.7 months instead of about 3 months. Using lower values for *h*_1_/*h*_2_ leads to more optimistic model predictions for the prospects of elimination. For the sensitivity analysis we shifted infectivity from primary to chronic infection while retaining a constant total infectivity. For a ratio of *h*_1_/*h*_2_ = 4.65 with *h*_1_ = 0.828 and *h*_2_ = 0.178 elimination can be achieved for smaller values of treatment uptake percentage and in a wider range of mixing parameter (*ω* ∈ [0.85, 1] and *ω* ∈ [0.58, 1] for τ* of 30% and 90%, respectively; see Fig 9). If we further shortened the duration of primary phase, the results would get even more favorable for elimination prospects. However, for a given *R*_0_ lowering *h*_1_/*h*_2_ would imply an increase in *λ*. Had we taken *h*_1_/*h*_2_ = 4.65 in our main analysis the value of *λ* would have had to be higher to correspond to a plausible range of *R*_0_. Taking both effects together (lowering ratio *h*_1_/*h*_2_ and increasing *λ*) would lead us back to similar results as described above, as there is a trade-off between the effects of these two parameters [11].

#### Treatment uptake during primary infection

**Fig 10:**
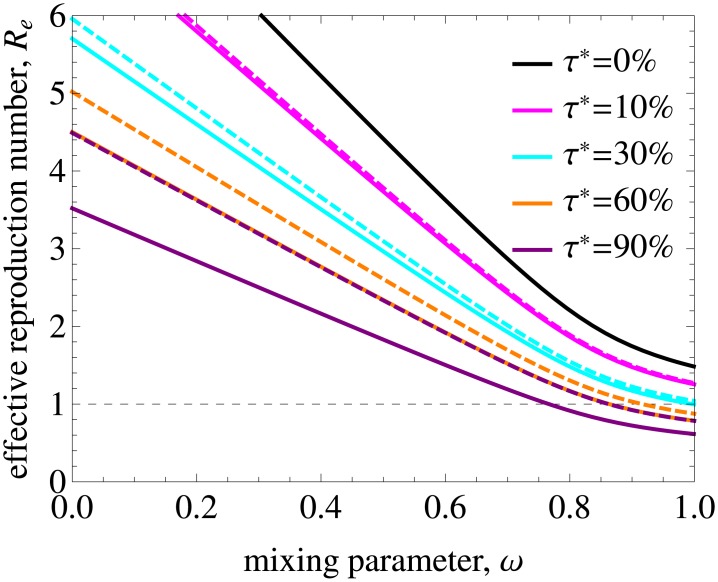
Effective reproduction number as a function of mixing and treatment uptake with and without uptake during the primary infection. The solid lines are repeated from Fig 6A and correspond to the annual treatment uptake percentage, τ*. The dashed lines were obtained when treatment uptake during the primary infection was set to 0 for all risk groups. As expected, elimination is more difficult to achieve in this case and the impact of not treating primary infection increases with increasing treatment uptake.

So far we have assumed that treatment uptake is independent of infection stage. In Fig 10 we relax this assumption by setting treatment uptake rate during the primary infection to 0 for all risk groups. As expected, elimination is more difficult to achieve in this case, especially if treatment uptake rate is high (compare the dashed and solid lines in Fig 10).

#### Population stratification

In our analysis the number of risk groups, *m*, and the respective initial fractions, *q*_*l*_, where *l* = 1, …, *m*, can be chosen arbitrarily. This choice then determines estimates of *c*_*l*_, *l* = 1, …, *m*. To illustrate the effect of population stratification on the effective reproduction number we compared our baseline parameter values to the extreme case when all initial population fractions are equal (*q*_*l*_ = 1/6 for *l* = 1, …, *m* = 6), see S8 Fig. The equal stratification leads to the situation closer to the homogeneous population because it smoothens out differences in partner change rates in the 6 risk groups. In this case *R*_*e*_ is below 1 for a treatment uptake above 10% independently of the mixing pattern.

## Discussion

We investigated how heterogeneity in sexual behaviour impacts on model predictions concerning the effects of ART on endemic HIV prevalence and on the prospects of eliminating HIV from a population. Heterogeneity in the model depended on two parameters, namely the variance in the rate of partner change and the mixing between subpopulations with different risk levels. This allowed us to compare populations that have the same average partner change rates, but differ in the way partnerships are distributed in the population.

We found that both parameters had a large influence on the basic reproduction number and endemic prevalence before ART. HIV would not have been able to spread in populations with proportionate mixing and a low level of overdispersion in the distribution of numbers of partners. For realistic MSM populations, where some degree of assortativeness is always present, *R*_0_ is above 1 and is higher if high risk individuals preferably mix with other high risk individuals. The distribution of infection across risk groups is skewed with high prevalence in small high risk subgroups. Moreover, this effect gets more pronounced as assortativeness of mixing increases.

The range of variances and any level of assortativeness in the model can reflect the range of MSM sexual behaviours found in Western societies. We used an average partner change rate estimated from MSM sexual behavior survey in the Netherlands. A different value for this parameter estimated from another data set (e.g. UK NATSAL data [51]) would lead to slightly different quantitative predictions but all qualitative conclusions for the dependence of the basic reproduction number on mixing and overdispersion in the distribution of numbers of partners would remain unchanged.

In the model, ART uptake is able to decrease the effective reproduction number below 1 and lead to HIV elimination. For some optimistic scenarios we found that an annual treatment uptake of at least 30% by all risk groups is necessary to eliminate HIV from populations with proportionate mixing. This uptake translates into a treatment coverage of at least 80% of all HIV infected individuals which is in line with the UNAIDS 90/90/90 treatment target to be reached by 2020. Thus we demonstrate that it may be possible to reach elimination for a realization of these objectives in relatively homogeneous populations regardless of heterogeneity in uptake of test-and-treat by risk group, but not in populations with strong heterogeneity in sexual behavior and mixing. For other types of mixing patterns which are more realistic even higher levels of coverage are necessary. For subpopulations with strongly assortative mixing, the model predicts that elimination with test-and-treat strategy is not feasible and additional interventions reducing the number of sexual partners and/or promoting condom use and PrEP uptake have to be applied. These conclusions agree with those of Dodd et al [52] who showed that test-and-treat in a hyper-endemic African setting generates a smaller impact in a population with heterogeneous risk distribution and assortative mixing than in that with random mixing assuming the intervention is implemented in the same way in both populations. In our model this happens because a high risk core group with a lot of within group mixing will enable persistent transmission within this small group. However, in the presence of heterogeneity in ART uptake, elimination will be easier to achieve when the subpopulation with highest risk behavior is tested and treated more often than the rest of the population. HIV elimination will be easier to achieve as well if the infectivity of primary phase is lower and its duration is shorter as was proposed by Bellan et al [49]. When HIV cannot be fully eliminated, the reduction in endemic prevalence will be still significant. Even if a population is almost fully assortative, we expect the reduction to be of about 57% for an annual treatment uptake of 30% by all risk groups and baseline infectivities.

For many populations we have some knowledge of rates of partner change, or at least numbers of partners reported in a given time period, but usually we have much less information on mixing patterns. Nevertheless, we are interested in using mathematical models based on available data for projecting effects of interventions into the future. We therefore need to be aware of the strong influence of heterogeneity on model outcomes. While it is probably not realistic to gather detailed information on sexual network structure for large populations, our modelling approach offers other ways of extracting information on behavioural heterogeneity from existing data. Linking the impact of behavioural heterogeneity with epidemic outcome distributions in a Lorenz curve allows estimation of the parameters which control heterogeneity by fitting the model to the data based on Lorenz curve. This requires collecting data with individual linkage of sexual behaviour and infection status, as was collected for chlamydia infection in the large UK NATSAL studies [51]. Earlier, comparing such data with outcomes for several individual based models was used to compare the ability of different models to correctly reproduce underlying sexual behaviour networks from population level parameters [47].

As with all models, also our approach has limitations. Our model is deterministic and thus it does not take stochastic effects into account. While stochastic fluctuations can play a role in real populations, where one superspreading individual can have large influence on transmission dynamics, here we were interested in mean effects that can occur over a long time period. The model is simplistic in some aspects of diagnosis, testing and ART uptake. In particular, there is no distinction between HIV-infected individuals who diagnosed and undiagnosed. First, second and third line treatments are not incorporated in the model explicitly. The parameters of the model are based on self-reported sexual behavior which might not be a true reflection. Sexual risk behaviour is stratified into a constant number of levels, and individuals remain in the same strata during their life time. That changes of risk behaviour of individuals in various phases of their lives can be important for HIV dynamics has been highlighted in recent work by Alam et al; Henry et al [19–22, 24, 25]. Also, our model does not take partnership duration into account, and therefore does not allow for long term concurrent relationships, which have been debated as a possible driver of HIV transmission in sub Saharan African heterosexual populations [53–55]. Therefore, our model is more amenable for describing HIV epidemics in MSM populations where concurrent partnerships are less influential for HIV transmission dynamics [56]. The data sets were used as an example to choose plausible parameter values, but we did not attempt to formally fit the model to a comprehensive set of available data. A more data driven approach to analyzing the HIV epidemic among MSM under ART in the Netherlands, for whom the degree of mixing has not been measured directly, is a focus of our ongoing work.

To conclude, we developed a modeling approach to investigate the impact of various mixing patterns in a population stratified by rates of partner change on the basic reproduction number, treatment effects and prospects of elimination. Our analysis revealed that both the variance in the rate of partner change and mixing between subpopulations with different risk levels have a large influence on endemic prevalence before introduction of ART and on possible long term effectiveness of ART. The developed framework offers a way of extracting information on behavioral heterogeneity from existing data, particularly assortativeness of a population, which would be otherwise very hard to measure in a population survey. Such information on behavioural heterogeneity should be taken into account when setting intervention goals and for analysis of cost-effectiveness of test-and-treat programmes in populations of MSM.

## Supporting Information

S1 Text Computation of the effective reproduction number. (PDF)

S1 Fig Time-dependent behavior of the model variables for the default parameter values without ART. (PDF)

S2 Fig Time-dependent behavior of the model variables for the default parameter values with ART. (PDF)

S3 Fig (A) Cumulative distribution function for the Weibull distribution in the partner change rate and the empirical cumulative frequency distribution obtained from WPF sexual behavior data for MSM in the Netherlands. (B) Distributions in the partner change rate used in the analysis have the same mean rate estimated from the WPF data and different variances. (C) Probability density function for the Weibull distribution in the partner change rate fitted to the WPF data histogram by maximum likelihood method.
(PDF)

S4 Fig Cumulative distribution function for the Weibull (A) and Gamma (B) distributions in the partner change rate and the empirical distribution obtained from the WPF data. (PDF)

S5 Fig Reduction in population size due to AIDS-related mortality. (PDF)

S6 Fig Dependence of the Lorenz curves on treatment uptake and mixing. (PDF)

S7 Fig *R*_*e*_ for heterogeneous uptake of testing and treatment. (PDF)

S8 Fig Effect of the population stratification on the effective reproduction number. (A) Stratification used throughout the main text. (B) Equal stratification into the 6 risk groups. (C) Effective reproduction number as a function of mixing and treatment uptake for the population stratification used in A and in B.
(PDF)

S1 Table Description of the parameters of the model and their baseline values. (PDF)

S2 Table The variance, the shape and the scale parameters of the distributions used in the analysis and the respective partner change rates in the 6 risk groups. (PDF)
