# Supplementary material for: Impact of Heterogeneity in Sexual Behavior on Effectiveness in Reducing HIV Transmission with Test-and-Treat Strategy
Source: PLoS Comput Biol. 2016 Aug 1;12(8):e1005012. doi: 10.1371/journal.pcbi.1005012 (PMC4968843; doi:10.1371/journal.pcbi.1005012)
Supplement: S1 Text — (PDF) [file pcbi.1005012.s001.pdf]

Impact of heterogeneity in sexual behavior on effectiveness in reducing HIV transmission with test-and-treat strategy

Supplementary Material: Computation of the effective reproduction number

Ganna Rozhnova[1], *Maarten F. Schim van der Loeff, Janneke C. M. Heijne and Mirjam E. Kretzschmar*

There are several methods that can be used to calculate the effective reproduction number, R_e, and the basic reproduction number, R_0, for a population with heterogeneity in sexual activity and any mixing pattern [1]. We used the method known in the literature as the next-generation matrix approach. The discussion of this standard method in the context of compartmental epidemiological models such as the SIR model or other related models of infectious diseases can be found in [2, 3]. Here we briefly revise the steps of the calculation of R_e for our HIV model with homogeneous treatment uptake

$$\frac{\mathrm{d}S_l}{\mathrm{d}t} = \mu N_l^0 - \mu S_l - J_l S_l, \tag{1}$$

$$\frac{\mathrm{d}I_{l1}}{\mathrm{d}t} = J_l S_l - (\mu + \rho_1 + \tau) I_{l1} + \phi A_{l1}, \tag{2}$$

$$\frac{\mathrm{d}I_{lk}}{\mathrm{d}t} = \rho_{k-1} I_{l,k-1} - (\mu + \rho_k + \tau) I_{lk} + \phi A_{lk}, \tag{3}$$

$$\frac{\mathrm{d}A_{l1}}{\mathrm{d}t} = \tau I_{l1} - (\mu + \gamma_1 + \phi) A_{l1}, \tag{4}$$

$$\frac{\mathrm{d}A_{lk}}{\mathrm{d}t} = \tau I_{lk} + \gamma_{k-1} A_{l,k-1} - (\mu + \gamma_k + \phi) A_{lk}, \tag{5}$$

where $k = 2, \ldots, n$ and $l = 1, \ldots, m$. S_l, I_{lk} and A_{lk} denote the number of susceptible, infected and treated individuals in stage k and risk group l. The description of the parameters of the model is given in Table 1. The computation of R_0 follows the same steps, with the only difference that the starting point is the system of differential equations without the treated population. For heterogeneous uptake by risk group τ has be substituted by τ_l in Eq. (1)-(5) but the procedure for the computation of R_e is still the same.

We parametrized the model for the case of $m = 6$ risk groups considered previously in modeling dynamics of Hepatitis B virus in MSM populations in the UK and the Netherlands [4, 5, 6, 7, 8]. From these studies we adopted the initial fractions of the population in the 6 risk groups, q_l, where $q_l \leq 1$ for $l = 1, \ldots, 6$ and $\sum_{l=1}^{6} q_l = 1$. However, the calculation of R_e does not depend on the specific number of risk groups as far as this number is finite. We, therefore, describe the general calculation for m groups.

[1]corresponding author: G.Rozhnova@umcutrecht.nl

We start by calculating the Jacobian matrix $\mathbf{J}$ of Eqs. (1)-(5) where the population size in group l is expressed as the sum of all other compartments, $N_l = S_l + \sum_{k=1}^{n} (I_{lk} + A_{lk})$, evaluated at the infection free equilibrium

$$S_l^* = q_l N_0 = N_l^0, \quad I_{lk}^* = A_{lk}^* = 0, \tag{6}$$

where $l = 1, \ldots, m$ and $k = 1, \ldots, n$.

As demonstrated in [3], the Jacobian $\mathbf{J}$ can be written as a sum of two matrices, a matrix of transmissions $\mathbf{T}$ and a matrix of transitions $\mathbf{\Sigma}$,

$$\mathbf{J} = \mathbf{T} + \mathbf{\Sigma}. \tag{7}$$

R_e then equals the dominant eigenvalue of the next generation matrix defined as follows [3]

$$\mathbf{K} = -\mathbf{T}\mathbf{\Sigma}^{-1}. \tag{8}$$

In the following we give explicit expressions for the matrices $\mathbf{\Sigma}$ and $\mathbf{T}$. The transition matrix $\mathbf{\Sigma}$ is a block diagonal matrix whose diagonal elements are identical submatrices $\bar{\mathbf{\Sigma}}_l = \bar{\mathbf{\Sigma}}$, $l = 1, \ldots, m$,

$$\mathbf{\Sigma} = \begin{pmatrix} \bar{\mathbf{\Sigma}}_1 & 0 & \ldots & 0 & 0 \\ 0 & \bar{\mathbf{\Sigma}}_2 & \ldots & 0 & 0 \\ \vdots & \vdots & \ddots & \vdots & \vdots \\ 0 & 0 & \ldots & \bar{\mathbf{\Sigma}}_{m-1} & 0 \\ 0 & 0 & \ldots & 0 & \bar{\mathbf{\Sigma}}_m \end{pmatrix}. \tag{9}$$

The identical diagonal elements $\bar{\mathbf{\Sigma}}$ are themselves block matrices composed from submatrices $\mathbf{\Pi}_k$ and $\mathbf{\Gamma}_k$ as follows

$$\bar{\mathbf{\Sigma}} = \begin{pmatrix} -\mu & 0 & 0 & 0 & \ldots & 0 & 0 \\ 0 & \mathbf{\Pi}_1 & 0 & 0 & \ldots & 0 & 0 \\ 0 & \mathbf{\Gamma}_1 & \mathbf{\Pi}_2 & 0 & \ldots & 0 & 0 \\ 0 & 0 & \mathbf{\Gamma}_2 & \mathbf{\Pi}_3 & \ldots & 0 & 0 \\ 0 & \vdots & \vdots & \vdots & \ddots & \vdots & \vdots \\ 0 & 0 & 0 & 0 & \ldots & \mathbf{\Pi}_{n-1} & 0 \\ 0 & 0 & 0 & 0 & \ldots & \mathbf{\Gamma}_{n-1} & \mathbf{\Pi}_n \end{pmatrix}. \tag{10}$$

The matrix $\bar{\mathbf{\Sigma}}$ contains n submatrices $\mathbf{\Pi}_k$ along the diagonal and $(n-1)$ submatrices $\mathbf{\Gamma}_k$ along the subdiagonal where

$$\mathbf{\Pi}_k = \begin{pmatrix} -\mu - \rho_k - \tau & \phi \\ \tau & -\mu - \gamma_k - \phi \end{pmatrix} \tag{11}$$

and

$$\mathbf{\Gamma}_k = \begin{pmatrix} \rho_k & 0 \\ 0 & \gamma_k \end{pmatrix}, \quad k = 1, \ldots, n. \tag{12}$$

Note that the transition matrix $\mathbf{\Sigma}$ given by Eq. (9) has a special form, i.e. diagonal, because we assumed that the disease progression, treatment uptake and dropping out of treatment are the same for all risk groups. When treatment uptake by risk groups differs, then $\mathbf{\Sigma}$ given by Eq. (9) is still block diagonal but its diagonal elements are not identical, $\bar{\mathbf{\Sigma}}_l \neq \bar{\mathbf{\Sigma}}$, $l = 1, \ldots, m$. The matrix $\bar{\mathbf{\Sigma}}_l$ has the form of Eq. (10), where in the expression for $\mathbf{\Pi}_k$ τ has to be substituted by τ_l.

This is not true for the transmission matrix $\mathbf{T}$ that depends on the mixing and heterogeneity in partner change rates. $\mathbf{T}$ consists of m^2 submatrices $\bar{\mathbf{T}}_{ll'}$ with $l, l' = 1, \ldots, m$ as follows

$$\mathbf{T} = \begin{pmatrix} \bar{\mathbf{T}}_{11} & \bar{\mathbf{T}}_{12} & \cdots & \bar{\mathbf{T}}_{1m} \\ \bar{\mathbf{T}}_{21} & \bar{\mathbf{T}}_{22} & \cdots & \bar{\mathbf{T}}_{2m} \\ \vdots & \vdots & \ddots & \vdots \\ \bar{\mathbf{T}}_{m1} & \bar{\mathbf{T}}_{m2} & \cdots & \bar{\mathbf{T}}_{mm} \end{pmatrix}, \tag{13}$$

where

$$\bar{\mathbf{T}}_{ll'} = \lambda c_l M_{ll'} \begin{pmatrix} 0 & -h_1 & -\epsilon & -h_2 & -\epsilon & \ldots & -h_n & -\epsilon \\ 0 & h_1 & \epsilon & h_2 & \epsilon & \ldots & h_n & \epsilon \\ 0 & 0 & 0 & 0 & 0 & 0 & 0 & 0 \\ \vdots & \vdots & \vdots & \vdots & \vdots & \vdots & \vdots & \vdots \\ 0 & 0 & 0 & 0 & 0 & 0 & 0 & 0 \end{pmatrix}. \tag{14}$$

Here $M_{ll'}$ is the element of the mixing matrix given by

$$M_{ll'} = \omega \frac{c_{l'} q_{l'}}{\sum\limits_{l''=1}^{m} c_{l''} q_{l''}} + (1 - \omega)\delta_{ll'}, \tag{15}$$

with

$$\delta_{ll'} = \begin{cases} 1, & \text{if } l = l' \\ 0, & \text{if } l \neq l'. \end{cases}$$

R_e is computed as the largest eigenvalue of the matrix $\mathbf{K}$ given by Eq. (8). This eigenvalue can be computed explicitly as a function of the parameters for arbitrary number of stages n and groups m because the matrix $\mathbf{K}$ has rank 1 and therefore the remaining eigenvalues equal zero. However, the expressions are too long for our default parameters and do not convey any insight, so we do not write them explicitly here.

References

[1] E. Vynnycky and R. White, *An introduction to infectious disease modelling.* Oxford: Oxford University Press, 2010.

[2] P. van den Driessche and J. Watmough, “Reproduction numbers and sub-threshold endemic equilibria for compartmental models of disease transmission,” *Mathematical Biosciences*, vol. 180, pp. 29–48, 2002.

[3] O. Diekmann, J. A. Heesterbeek, and M. G. Roberts, “The construction of next-generation matrices for compartmental epidemic models,” *Journal of the Royal Society Interface*, vol. 7, pp. 873–885, 2010.

[4] J. R. Williams, D. J. Nokes, G. F. Medley, and R. M. Anderson, “The transmission dynamics of hepatitis B in the UK: a mathematical model for evaluating costs and effectiveness of immunization programmes,” *Epidemiology & Infection*, vol. 116, pp. 71–89, 1996.

[5] R. M. Anderson, G. F. Medley, and D. J. Nokes, “Preliminary analyses of the predicted impacts of various vaccination strategies on the transmission of the hepatitis B virus,” in *Proceedings of the conference on the control of hepatitis B: the role of prevention in adolescence*, (London), pp. 95–130, 1991.

[6] M. Xiridou, R. van Houdt, S. Hahné, R. Coutinho, J. van Steenbergen, and M. Kretzschmar, “Hepatitis B vaccination of men who have sex with men in the Netherlands: should we vaccinate more men, younger men or high-risk men?,” *Sexually Transmitted Infections*, vol. 89, pp. 666–671, 2013.

[7] M. Kretzschmar, G. A. de Wit, L. J. M. Smits, and M. J. W. van de Laar, “Vaccination against hepatitis B in low endemic countries,” *Epidemiology & Infection*, vol. 128, pp. 229–244, 2002.

[8] M. Kretzschmar, M.-J. Mangen, M. van de Laar, and A. de Wit, “Model based analysis of hepatitis B vaccination strategies in the Netherlands,” *Vaccine*, vol. 27, pp. 1254–1260, 2009.
