# Supplementary material for: Impact of Heterogeneity in Sexual Behavior on Effectiveness in Reducing HIV Transmission with Test-and-Treat Strategy
Source: PLoS Comput Biol. 2016 Aug 1;12(8):e1005012. doi: 10.1371/journal.pcbi.1005012 (PMC4968843; doi:10.1371/journal.pcbi.1005012)
Supplement: S1 Fig — (PDF) [file pcbi.1005012.s002.pdf]

Time-dependent behavior of the model variables for the default parameter values without ART. The results are for the total number of susceptible individuals, $S(t)$, total number of infected individuals, $I(t)$, total population size, $N(t)$, and population sizes of the lowest, $N_1(t)$, and highest, $N_6(t)$, risk groups. Parameter values are as in S1 Table.

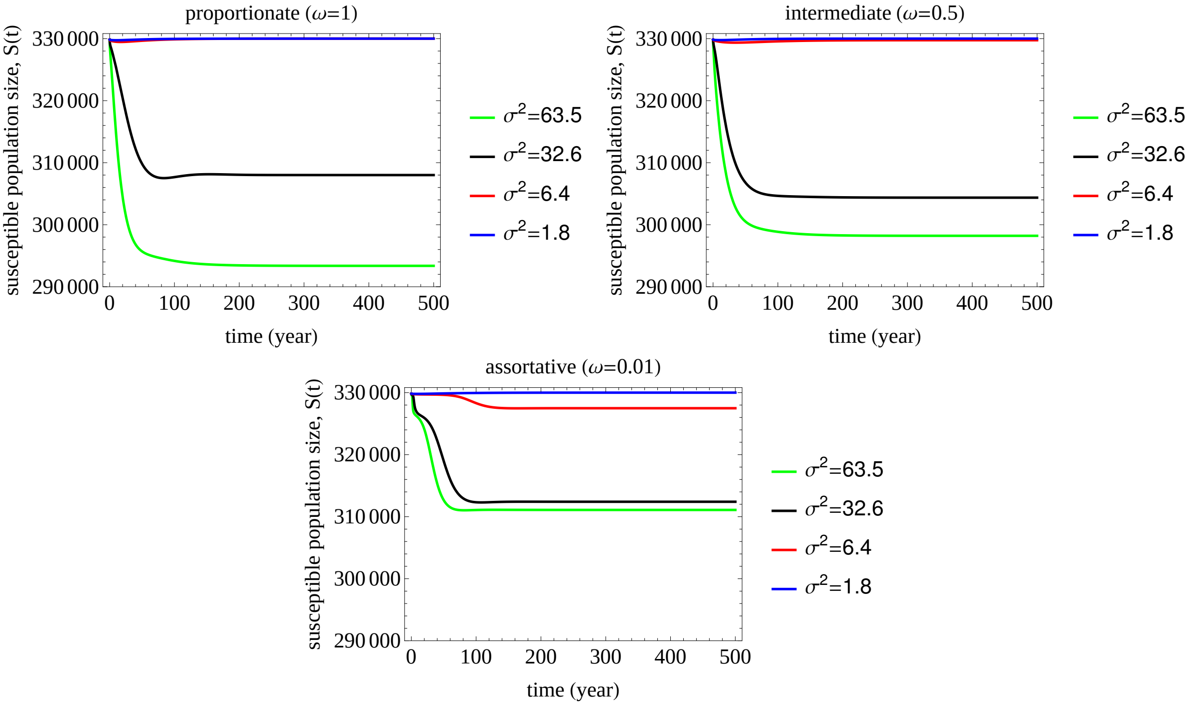

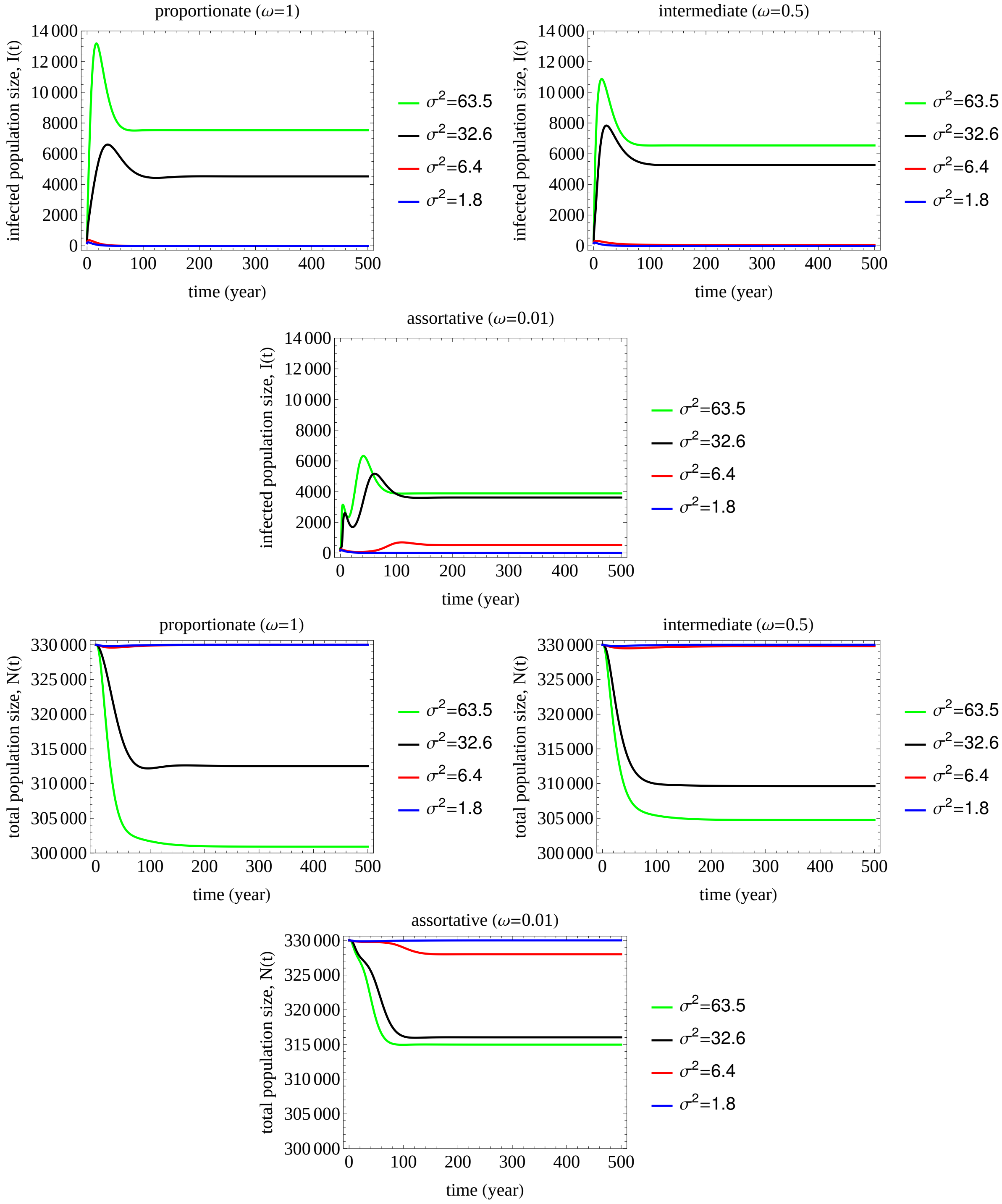
proportionate (ω=1)
intermediate (ω=0.5)
assortative (ω=0.01)
infected population size, I(t)
total population size, N(t)
time (year)
σ2=63.5
σ2=32.6
σ2=6.4
σ2=1.8

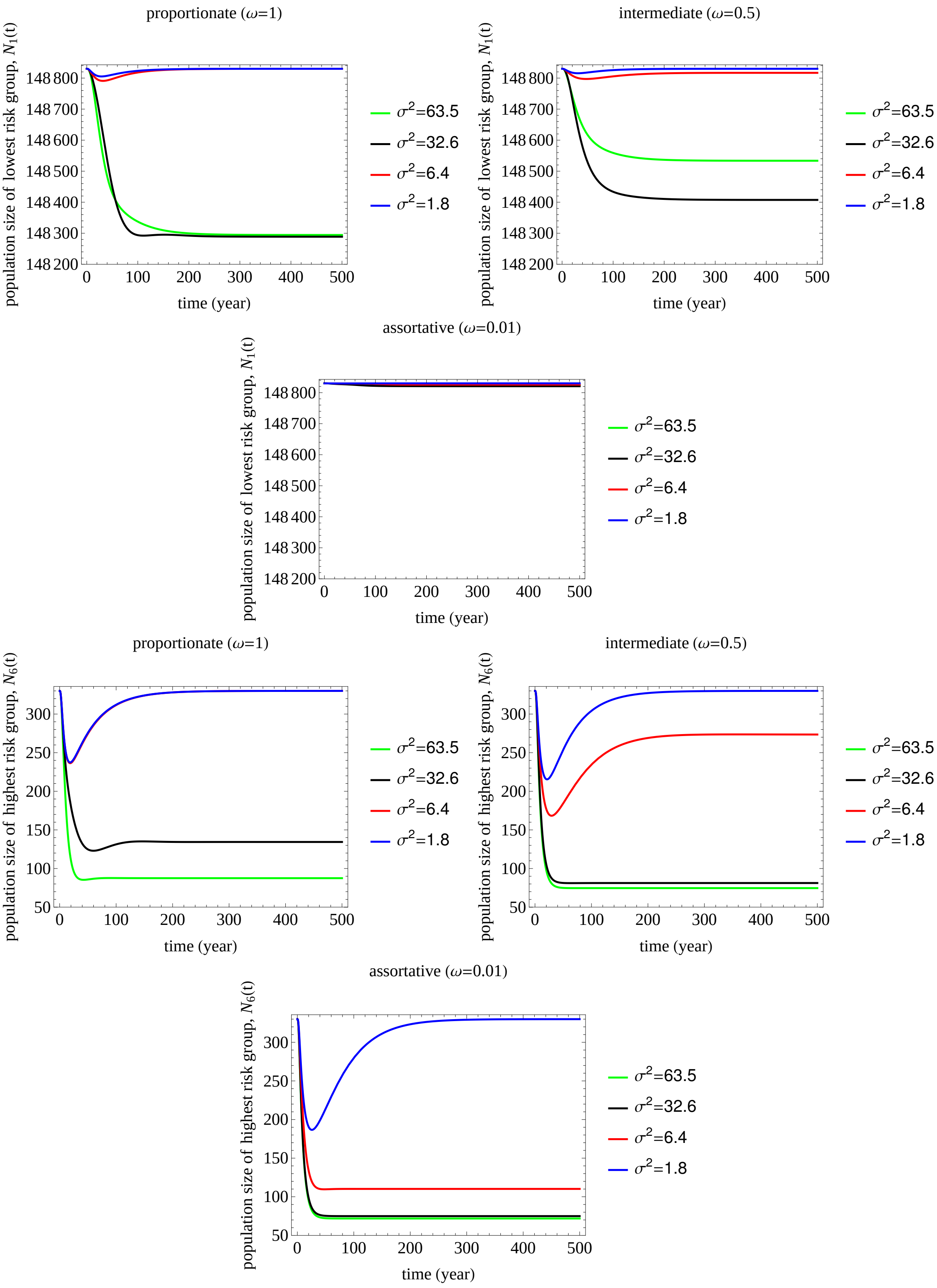
proportionate (ω=1)
intermediate (ω=0.5)
assortative (ω=0.01)
population size of lowest risk group, N1(t)
population size of highest risk group, N6(t)
time (year)
σ2=63.5
σ2=32.6
σ2=6.4
σ2=1.8
148 200
148 300
148 400
148 500
148 600
148 700
148 800
50
100
150
200
250
300
0
100
200
300
400
500
