# Supplementary material for: Impact of Heterogeneity in Sexual Behavior on Effectiveness in Reducing HIV Transmission with Test-and-Treat Strategy
Source: PLoS Comput Biol. 2016 Aug 1;12(8):e1005012. doi: 10.1371/journal.pcbi.1005012 (PMC4968843; doi:10.1371/journal.pcbi.1005012)
Supplement: S2 Fig — (PDF) [file pcbi.1005012.s003.pdf]

Time-dependent behavior of the model variables for the default parameter values with ART. The results are for the total number of susceptible individuals, $S(t)$, total number of untreated infected individuals, $I(t)$, total number of treated individuals, $A(t)$, total population size, $N(t)$, and population sizes of the lowest, $N_1(t)$, and highest, $N_6(t)$, risk groups. The annual treatment uptake percentage, τ^*, is 80% and the rest of parameter values are as in S1 Table.

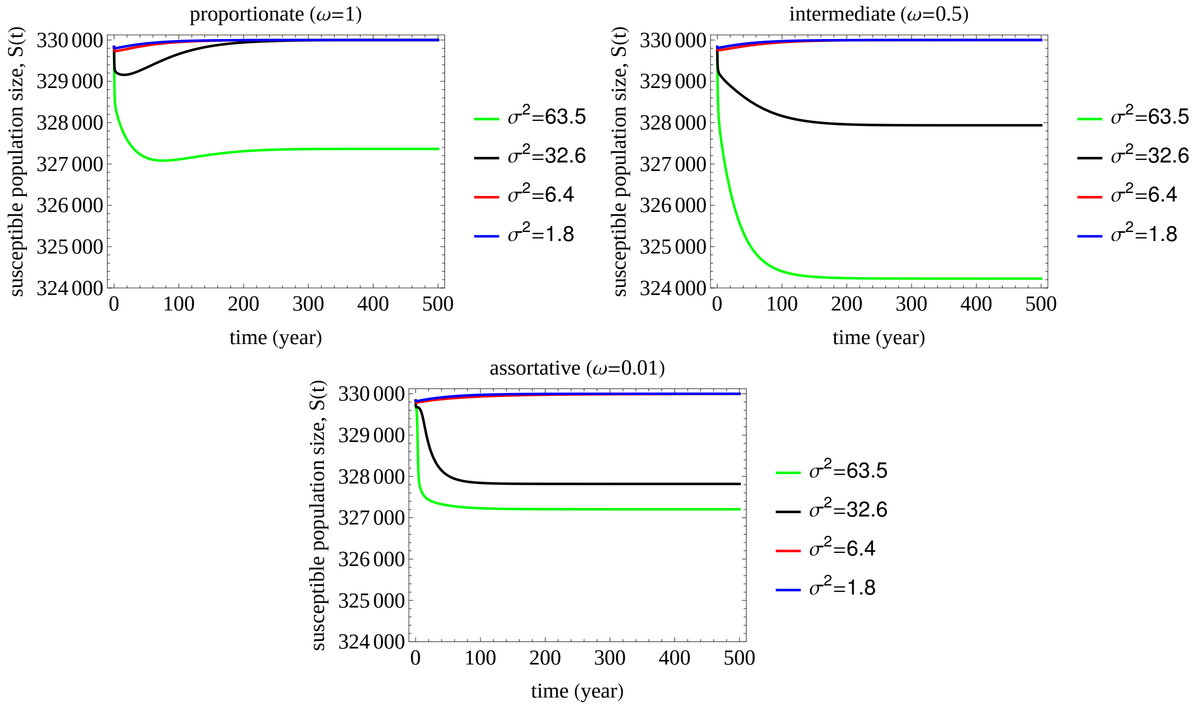

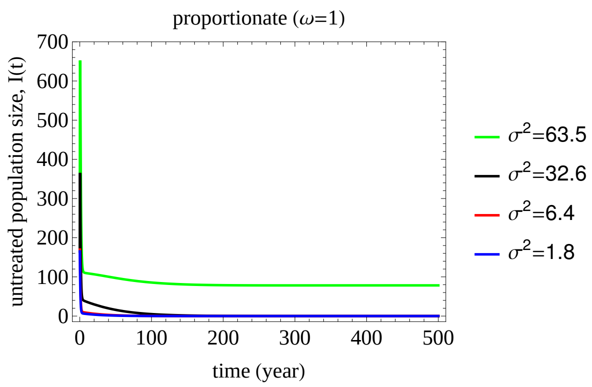
proportionate (ω=1)
untreated population size, I(t)
0
100
200
300
400
500
600
700
time (year)
0
100
200
300
400
500
σ²=63.5
σ²=32.6
σ²=6.4
σ²=1.8

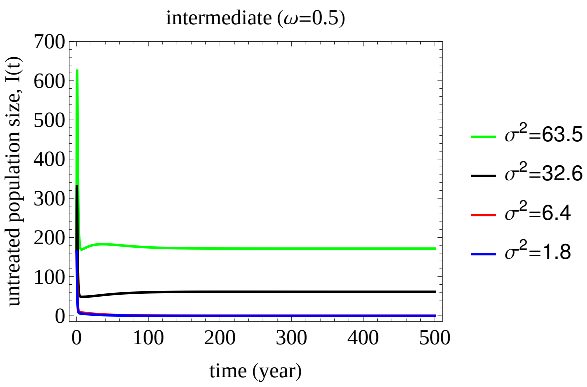
intermediate (ω=0.5)
untreated population size, I(t)
0
100
200
300
400
500
600
700
time (year)
0
100
200
300
400
500
σ²=63.5
σ²=32.6
σ²=6.4
σ²=1.8

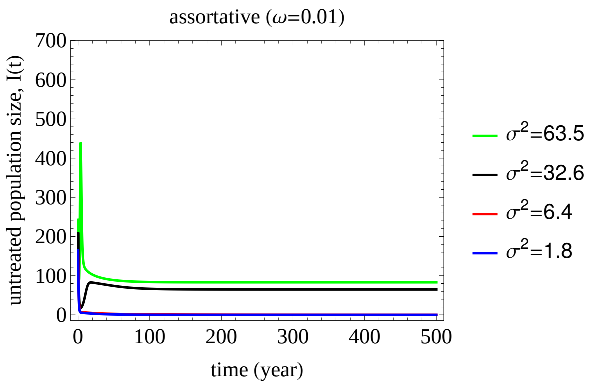
assortative (ω=0.01)
untreated population size, I(t)
0
100
200
300
400
500
600
700
time (year)
0
100
200
300
400
500
σ²=63.5
σ²=32.6
σ²=6.4
σ²=1.8

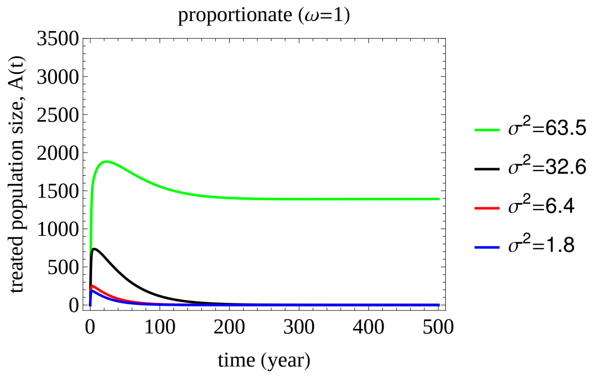
proportionate (ω=1)
treated population size, A(t)
0
500
1000
1500
2000
2500
3000
3500
time (year)
0
100
200
300
400
500
σ²=63.5
σ²=32.6
σ²=6.4
σ²=1.8

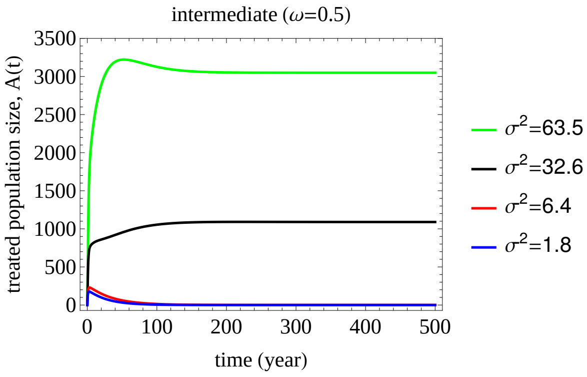
intermediate (ω=0.5)
treated population size, A(t)
0
500
1000
1500
2000
2500
3000
3500
time (year)
0
100
200
300
400
500
σ²=63.5
σ²=32.6
σ²=6.4
σ²=1.8

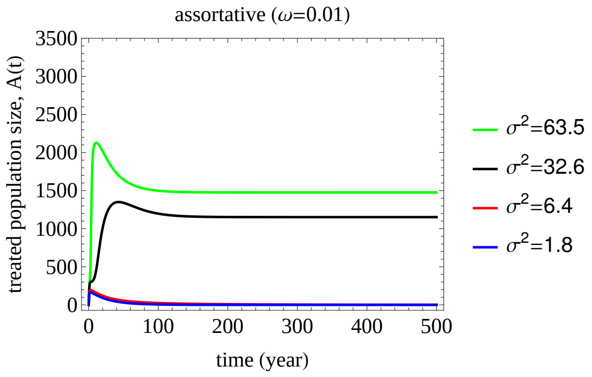
assortative (ω=0.01)
treated population size, A(t)
0
500
1000
1500
2000
2500
3000
3500
time (year)
0
100
200
300
400
500
σ²=63.5
σ²=32.6
σ²=6.4
σ²=1.8

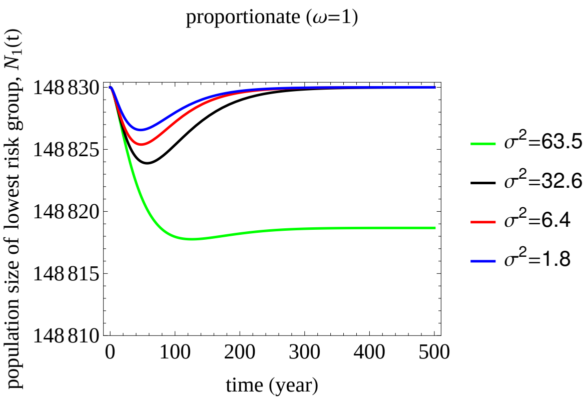
proportionate (ω=1)
population size of lowest risk group, N1(t)
148 830
148 825
148 820
148 815
148 810
0 100 200 300 400 500
time (year)
σ2=63.5
σ2=32.6
σ2=6.4
σ2=1.8

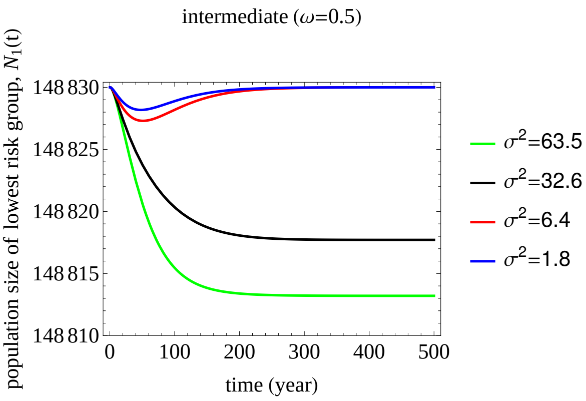
intermediate (ω=0.5)
population size of lowest risk group, N1(t)
148 830
148 825
148 820
148 815
148 810
0 100 200 300 400 500
time (year)
σ2=63.5
σ2=32.6
σ2=6.4
σ2=1.8

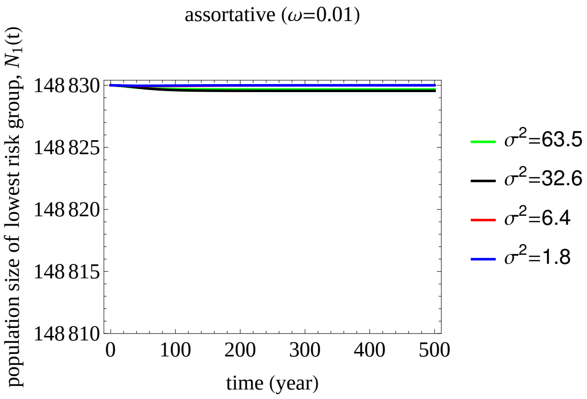
assortative (ω=0.01)
population size of lowest risk group, N1(t)
148 830
148 825
148 820
148 815
148 810
0 100 200 300 400 500
time (year)
σ2=63.5
σ2=32.6
σ2=6.4
σ2=1.8

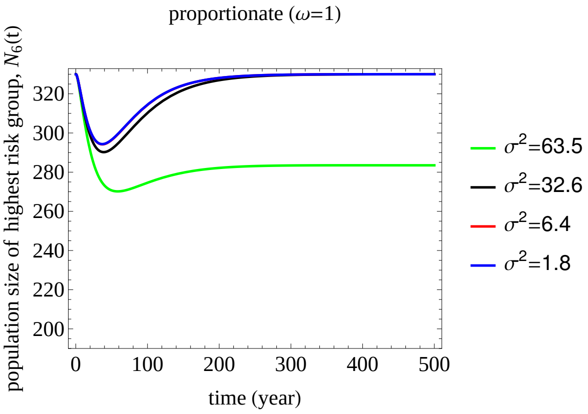
proportionate (ω=1)
population size of highest risk group, N6(t)
320
300
280
260
240
220
200
0 100 200 300 400 500
time (year)
σ2=63.5
σ2=32.6
σ2=6.4
σ2=1.8

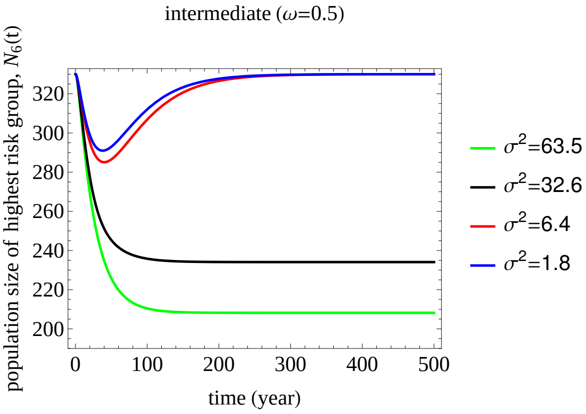
intermediate (ω=0.5)
population size of highest risk group, N6(t)
320
300
280
260
240
220
200
0 100 200 300 400 500
time (year)
σ2=63.5
σ2=32.6
σ2=6.4
σ2=1.8

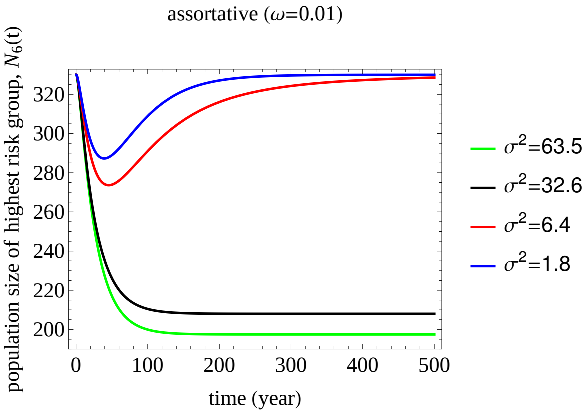
assortative (ω=0.01)
population size of highest risk group, N6(t)
320
300
280
260
240
220
200
0 100 200 300 400 500
time (year)
σ2=63.5
σ2=32.6
σ2=6.4
σ2=1.8

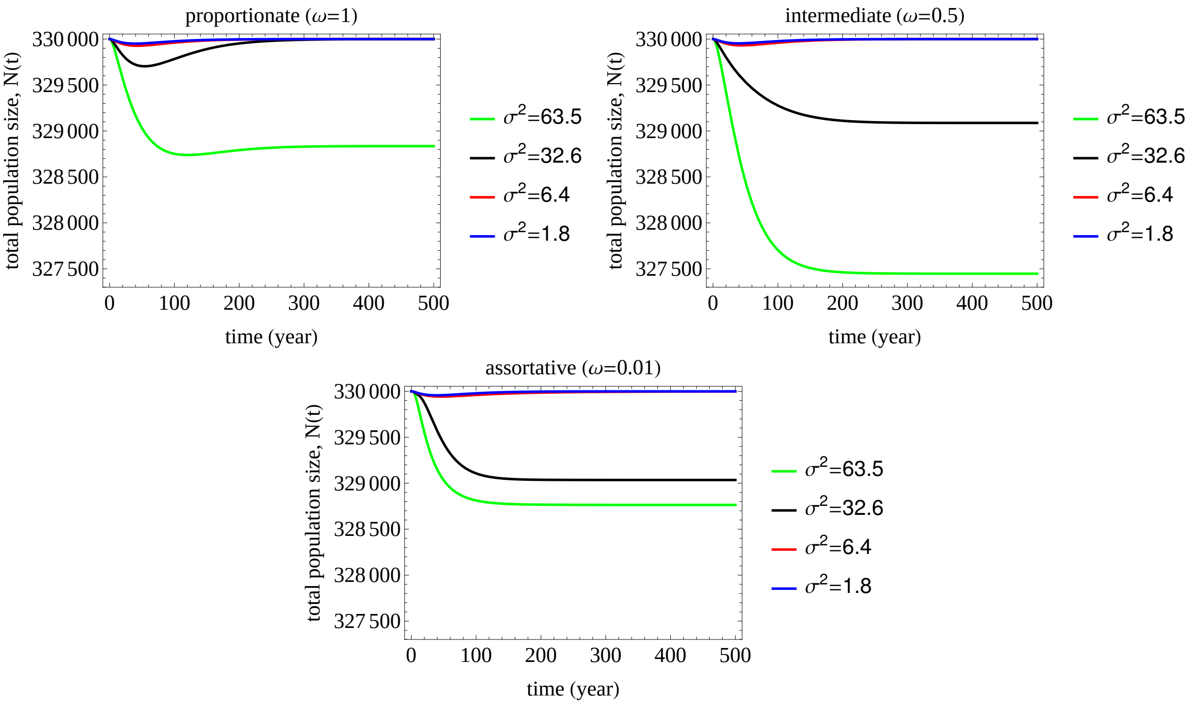
proportionate (ω=1)
intermediate (ω=0.5)
assortative (ω=0.01)
total population size, N(t)
time (year)
330 000
329 500
329 000
328 500
328 000
327 500
0
100
200
300
400
500
σ^2=63.5
σ^2=32.6
σ^2=6.4
σ^2=1.8
