# Supplementary material for: Impact of Heterogeneity in Sexual Behavior on Effectiveness in Reducing HIV Transmission with Test-and-Treat Strategy
Source: PLoS Comput Biol. 2016 Aug 1;12(8):e1005012. doi: 10.1371/journal.pcbi.1005012 (PMC4968843; doi:10.1371/journal.pcbi.1005012)

(A) The cumulative distribution function for the Weibull distribution in the partner change rate and the empirical cumulative frequency distribution obtained from WPF sexual behavior data for MSM in the Netherlands. The dashed lines indicate the intervals defined by the initial fractions of the population in the 6 risk groups, q_l, $l = 1, \ldots, 6$, per which mean rates of partner change were estimated.

(B) Distributions in the partner change rate used in the analysis have the same mean rate estimated from the WPF data and different variances. Shown are the cumulative distribution functions for the Weibull distribution with a mean of $c = 2.54$ partners per year and different variances, σ^2, obtained by varying the shape parameter, α, and the scale parameter, β. The Weibull distribution that best fits to the data is shown in black.

(C) Probability density function for the Weibull distribution in the partner change rate fitted to the WPF data histogram by maximum likelihood method. This panel is the same as Fig. 2A in the main text but it has wider ranges for the x and y axes to show more data points. For a better visualization the ranges of the x and y axes differ among the panels.

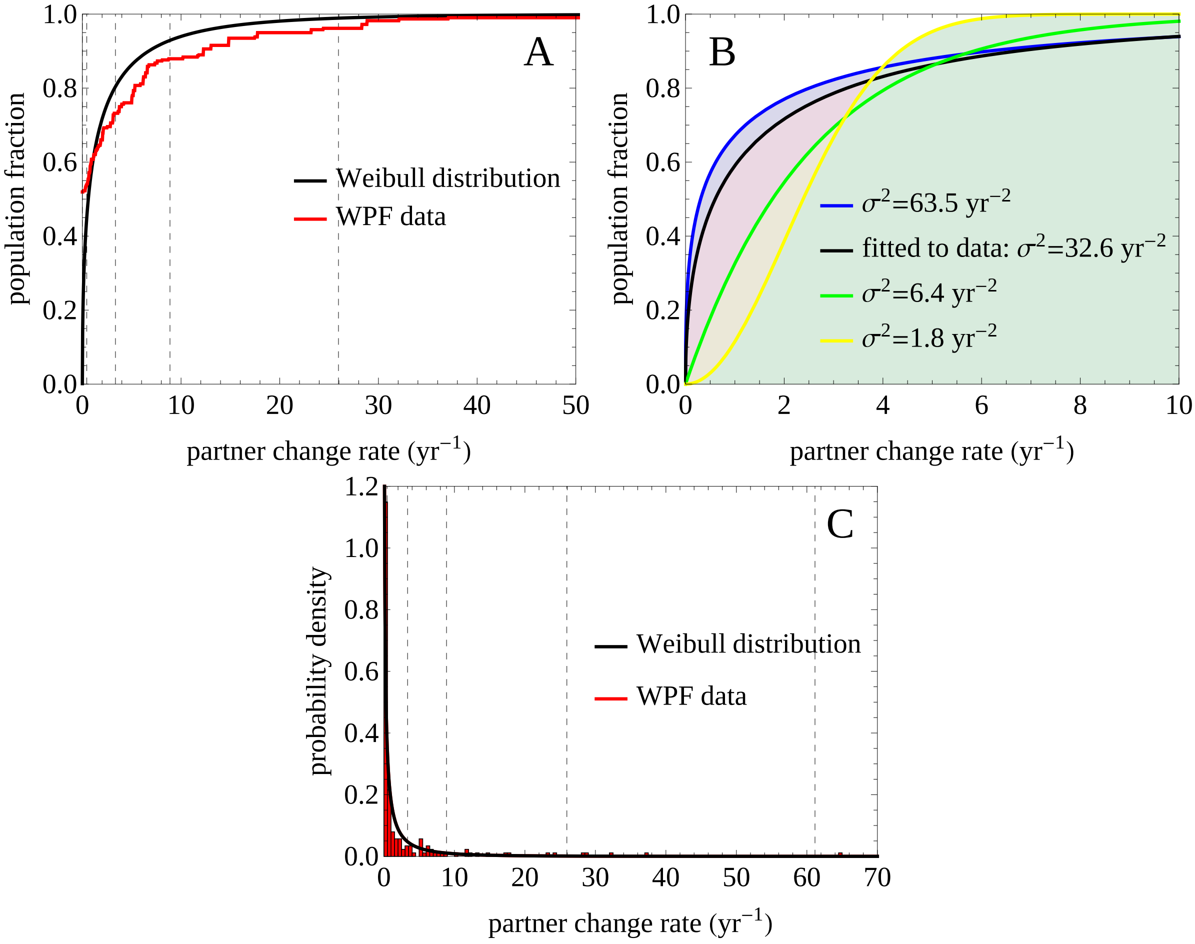

Supplement: S3 Fig — (A) Cumulative distribution function for the Weibull distribution in the partner change rate and the empirical cumulative frequency distribution obtained from WPF sexual behavior data for MSM in the Netherlands. (B) Distributions in the partner change rate used in the analysis have the same mean rate estimated from the WPF data and different variances. (C) Probability density function for the Weibull distribution in the partner change rate fitted to the WPF data histogram by maximum likelihood method. (PDF) [file pcbi.1005012.s004.pdf]
