# Supplementary material for: Impact of Heterogeneity in Sexual Behavior on Effectiveness in Reducing HIV Transmission with Test-and-Treat Strategy
Source: PLoS Comput Biol. 2016 Aug 1;12(8):e1005012. doi: 10.1371/journal.pcbi.1005012 (PMC4968843; doi:10.1371/journal.pcbi.1005012)

Cumulative distribution function for the Weibull and Gamma distributions in the partner change rate and the empirical distribution obtained from the WPF data. From the fitting procedure we obtain the estimates of the shape, α, and scale, β, parameters, the mean partner change rate, c, and the mean rates in the 6 risk groups, c_l, where $l = 1, \ldots, 6$. (A) Weibull distribution (panel A in S3 Fig): $\alpha = 0.497474$, $\beta = 1.25658$; $c = 2.53686$, $c_1 = 0.127469$, $c_2 = 1.4348$, $c_3 = 5.4394$, $c_4 = 14.1953$, $c_5 = 35.9707$, $c_6 = 81.5467$. (B) Gamma distribution: $\alpha = 0.356847$, $\beta = 8.50218$; $c = 3.03397$, $c_1 = 0.179485$, $c_2 = 2.2302$, $c_3 = 7.30403$, $c_4 = 15.2068$, $c_5 = 29.1232$, $c_6 = 49.1228$. All contact rates are in units yr^{-1}.

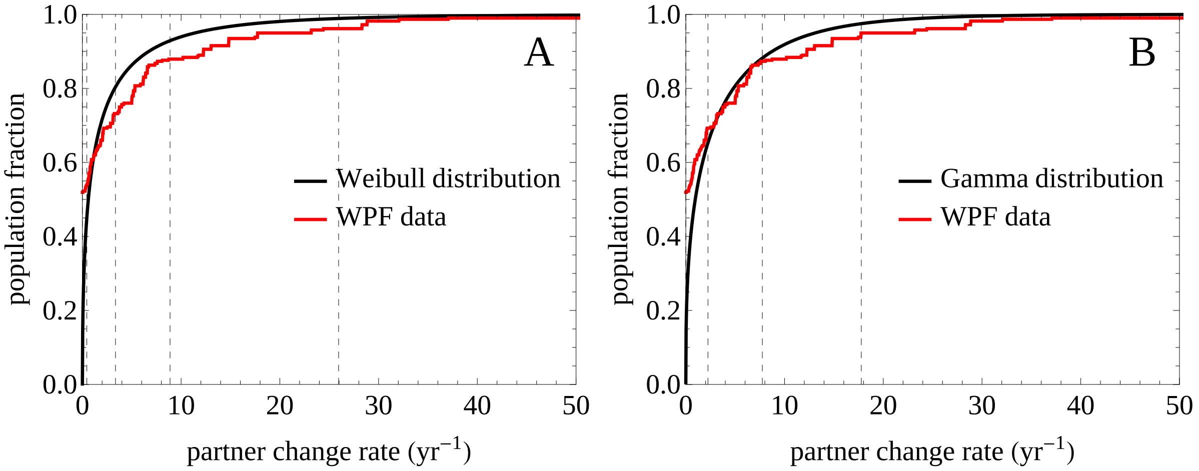

Supplement: S4 Fig — (PDF) [file pcbi.1005012.s005.pdf]
