# Supplementary material for: Impact of Heterogeneity in Sexual Behavior on Effectiveness in Reducing HIV Transmission with Test-and-Treat Strategy
Source: PLoS Comput Biol. 2016 Aug 1;12(8):e1005012. doi: 10.1371/journal.pcbi.1005012 (PMC4968843; doi:10.1371/journal.pcbi.1005012)
Supplement: S5 Fig — (PDF) [file pcbi.1005012.s006.pdf]

Reduction in population size due to AIDS-related mortality. Risk groups with larger prevalence experience larger mortality too which results in the nontrivial dependence of total prevalence on mixing and variance in the partner change rate shown in Fig. 4A in the main text. The panels show percentage reduction in sizes of the six risk groups and in the total population size in the steady state as a function of the variance in the partner change rate, σ^2, and mixing parameter, ω. The range of values for these parameters is the same as in Fig. 4A in the main text. Note that for a better visualization different panels have different ranges of the y-axis. The maximum reductions for the lowest risk group (group 1), the highest risk group (group 6) and the total population size in the whole rage of explored parameters do not exceed 0.37%, 78.22% and 8.9%, respectively. If we consider the largest value of σ^2 (blue bars) and proportionate mixing ($\omega = 1$) then the reduction in the size increases from the lowest risk group to the highest risk group (from 0.36% to 73.49%). For this variance (blue bars) the mortality increases for intermediate ($\omega = 0.5$) and assortative ($\omega = 0.01$) mixing too. The same type of pattern is observed if we fix σ^2 at the value estimated from the data (black bars) for all types of mixing. If the variance is low (green bars) epidemic persists when mixing is closer to assortative and goes extinct for proportionate mixing. The reduction is again the largest in the highest risk groups. For the lowest variance and any type of mixing pattern R_0 is smaller than 1 (see Fig. 3 in the main text). In this case the percentage reduction is 0% in all panels (yellow bars).

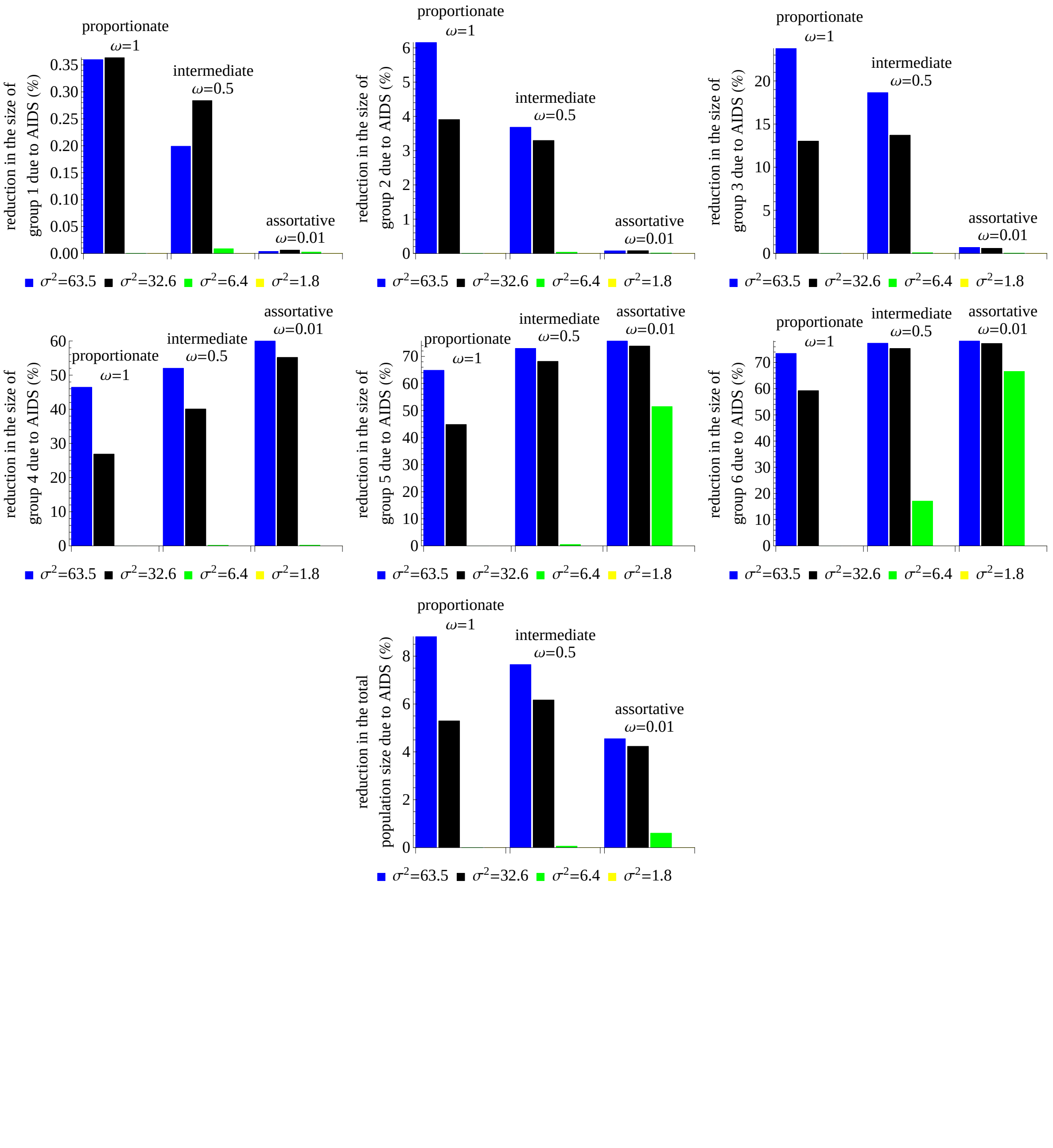
proportionate
ω=1
intermediate
ω=0.5
assortative
ω=0.01
reduction in the size of group 1 due to AIDS (%)
reduction in the size of group 2 due to AIDS (%)
reduction in the size of group 3 due to AIDS (%)
reduction in the size of group 4 due to AIDS (%)
reduction in the size of group 5 due to AIDS (%)
reduction in the size of group 6 due to AIDS (%)
reduction in the total population size due to AIDS (%)
σ2=63.5
σ2=32.6
σ2=6.4
σ2=1.8
