# Supplementary figures and images for: Impact of Heterogeneity in Sexual Behavior on Effectiveness in Reducing HIV Transmission with Test-and-Treat Strategy
Source: PLoS Comput Biol. 2016 Aug 1;12(8):e1005012. doi: 10.1371/journal.pcbi.1005012 (PMC4968843; doi:10.1371/journal.pcbi.1005012)

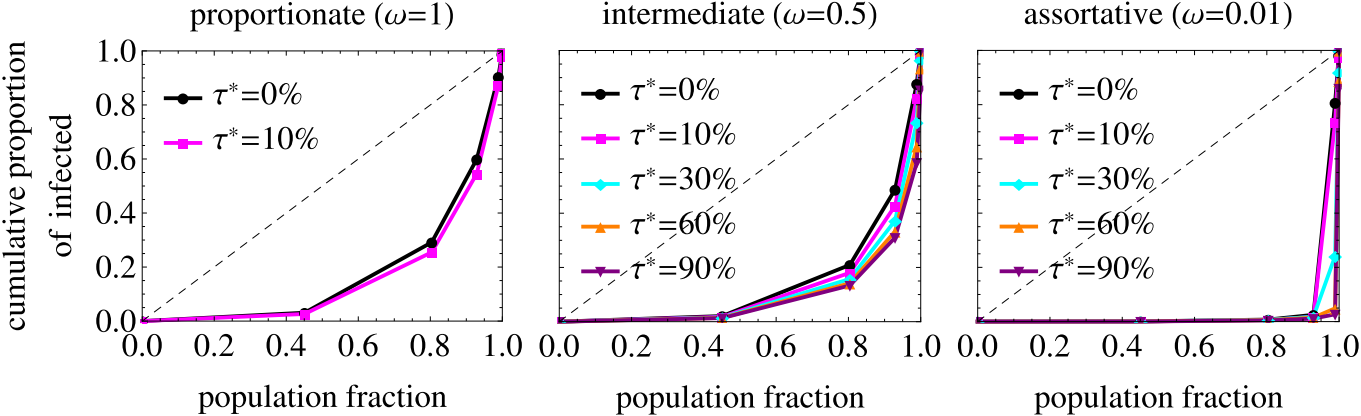

Supplement: S6 Fig — (PDF) [file pcbi.1005012.s007.pdf]
