# Supplementary material for: Impact of Heterogeneity in Sexual Behavior on Effectiveness in Reducing HIV Transmission with Test-and-Treat Strategy
Source: PLoS Comput Biol. 2016 Aug 1;12(8):e1005012. doi: 10.1371/journal.pcbi.1005012 (PMC4968843; doi:10.1371/journal.pcbi.1005012)

R_e **for heterogeneous uptake of testing and treatment.** We considered a situation in which the highest risk group has an annual uptake, τ_6^*, which is below or above the rest of the population with all τ_l^* equal for $l = 1, \ldots, 5$. The panels show R_e for $\tau_l^* = 10\%, 30\%, 60\%, 90\%$ ($l = 1, \ldots, 5$) and τ_6^* ranges between 10% and 90%. The dashed line is R_0 before ART. As expected, R_e has values above 1 (elimination is unfeasible) in a wider range of mixing parameter when ART uptake by highest risk individuals is smaller than by the rest of the population, and vice versa if they are tested and get treated more frequently.

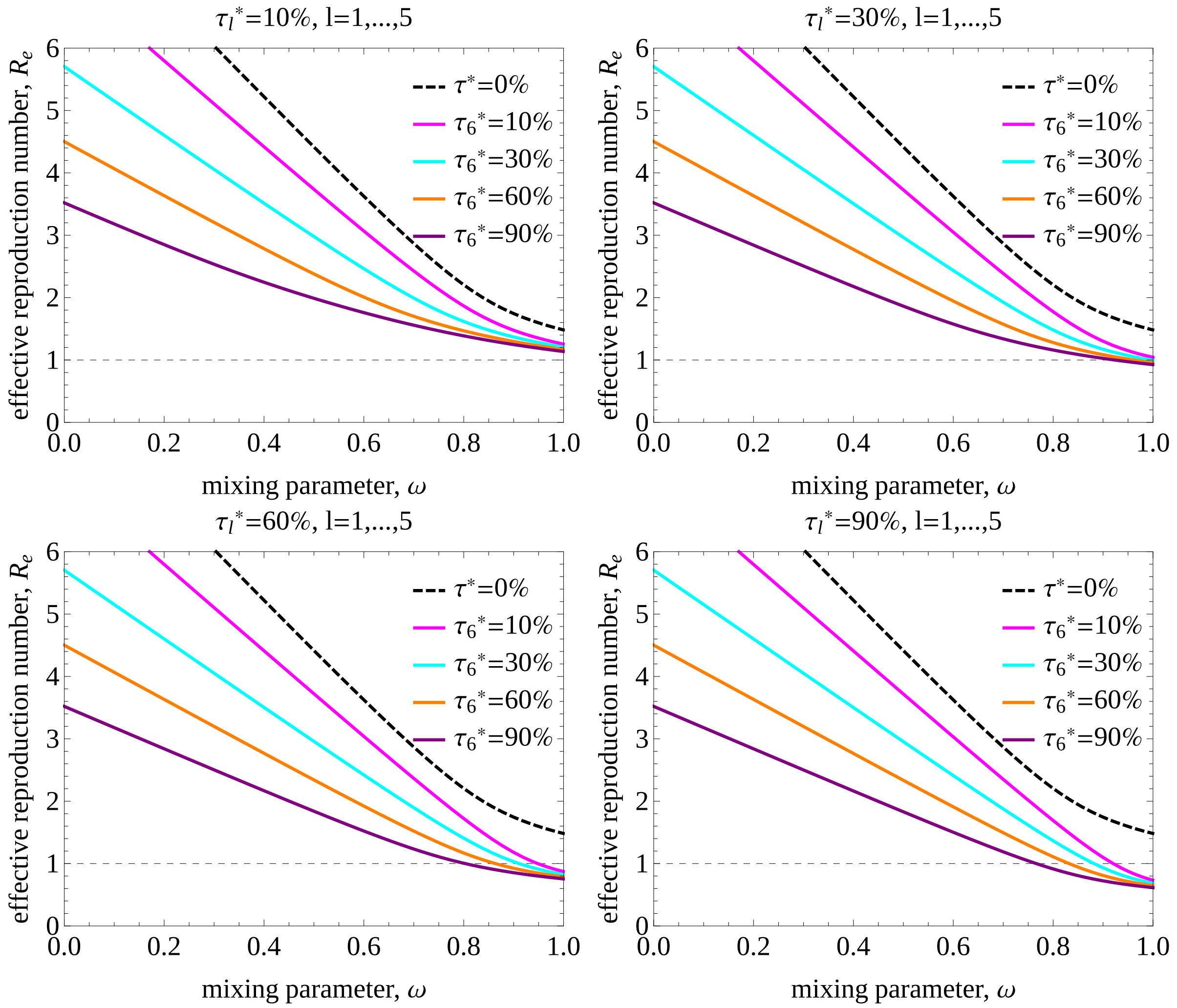

Supplement: S7 Fig — (PDF) [file pcbi.1005012.s008.pdf]
