# Supplementary material for: Impact of Heterogeneity in Sexual Behavior on Effectiveness in Reducing HIV Transmission with Test-and-Treat Strategy
Source: PLoS Comput Biol. 2016 Aug 1;12(8):e1005012. doi: 10.1371/journal.pcbi.1005012 (PMC4968843; doi:10.1371/journal.pcbi.1005012)

Effect of population stratification on the effective reproduction number. From the fitting procedure we obtain estimates of the shape, $\alpha = 0.497474$, and scale, $\beta = 1.25658$, parameters and the mean partner change rate, $c = 2.53686$ yr^{-1}. The mean rates of partner change in the 6 risk groups, c_l, where $l = 1, \ldots, 6$, depend on the choice of initial population fractions, q_l, where $l = 1, \ldots, 6$. (A) Stratification used throughout the main text (panel A in S3 Fig): initial population fractions $q_1 = 0.451$, $q_2 = 0.353$, $q_3 = 0.125$, $q_4 = 0.06$, $q_5 = 0.01$ and $q_6 = 0.001$; partner change rates $c_1 = 0.127469$, $c_2 = 1.4348$, $c_3 = 5.4394$, $c_4 = 14.1953$, $c_5 = 35.9707$, $c_6 = 81.5467$. (B) Equal stratification into the 6 risk groups: initial population fractions $q_l = 1/6$, where $l = 1, \ldots, 6$; partner change rates $c_1 = 0.0130195$, $c_2 = 0.10939$, $c_3 = 0.376127$, $c_4 = 0.994072$, $c_5 = 2.5408$, $c_6 = 11.1878$. All contact rates are in units yr^{-1}. As we see the stratification into equally sized groups leads to smaller differences between contact rates in different groups. (C) Effective reproduction number as a function of mixing and treatment uptake for the population stratification used in A (solid lines) and in B (dashed lines). The solid lines are repeated from Fig. 6A in the main text. For the equally stratified population the curves are below $R_e < 1$ for an annual treatment uptake percentage $\tau^* > 10\%$ independently of the mixing.

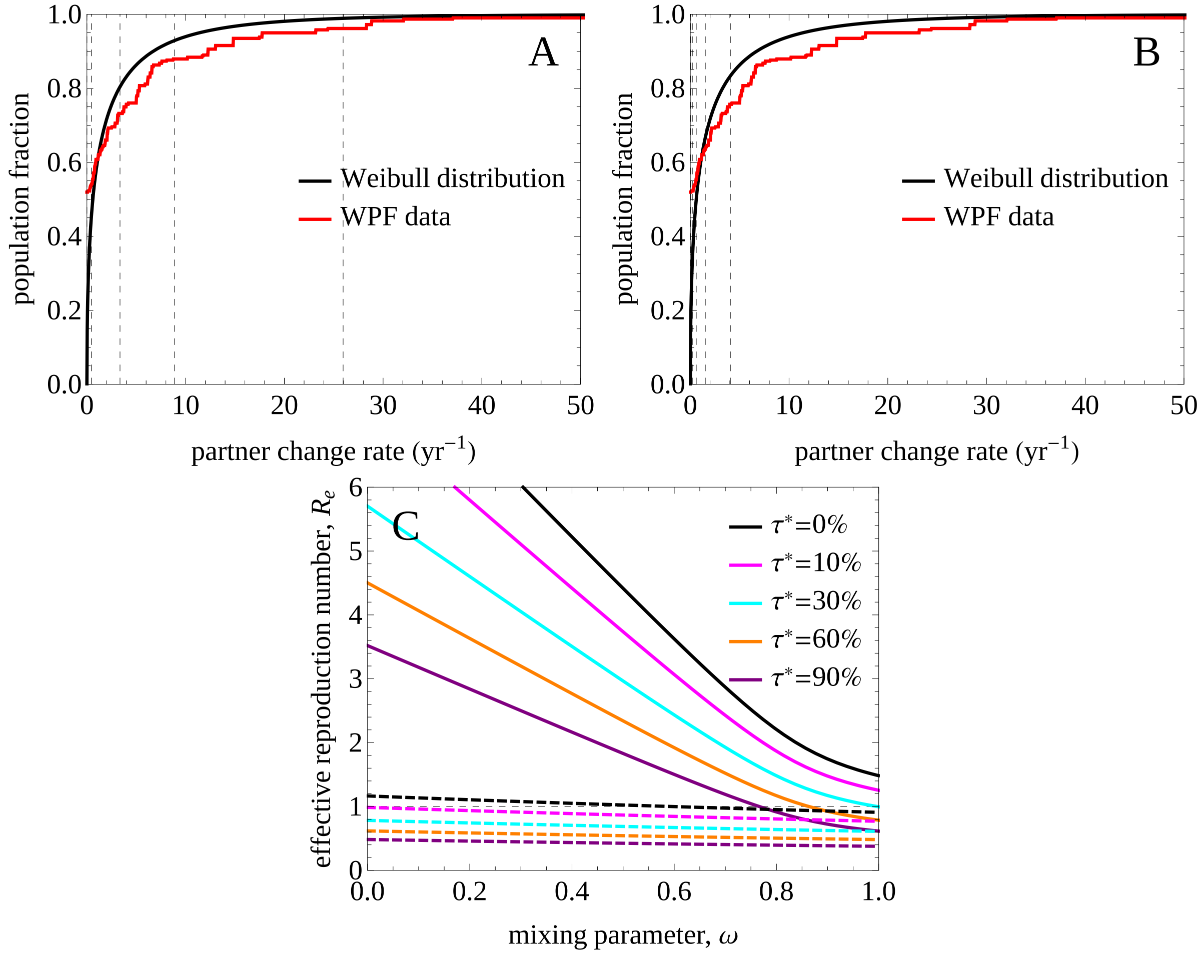

Supplement: S8 Fig — (A) Stratification used throughout the main text. (B) Equal stratification into the 6 risk groups. (C) Effective reproduction number as a function of mixing and treatment uptake for the population stratification used in A and in B. (PDF) [file pcbi.1005012.s009.pdf]
