# Supplementary material for: Impact of Heterogeneity in Sexual Behavior on Effectiveness in Reducing HIV Transmission with Test-and-Treat Strategy
Source: PLoS Comput Biol. 2016 Aug 1;12(8):e1005012. doi: 10.1371/journal.pcbi.1005012 (PMC4968843; doi:10.1371/journal.pcbi.1005012)
Supplement: S1 Table — (PDF) [file pcbi.1005012.s010.pdf]

Notation	Baseline value, unit	Description	Source
N_0	330 000	Initial population size	(1; 2)
$1/\mu$	45 years	Mean duration of sexual activity	(3; 4; 5)
μ	$1/45$ yr^{-1}	Rate of recruitment to sexually active population	(6)
ρ_k, $k = 1, 2, 3$	$\rho_1 = 1/0.271$ yr^{-1}<br>$\rho_2 = 1/8.31$ yr^{-1}<br>$\rho_3 = 1/1.184$ yr^{-1}	Rate of transition from stage k to stage $k+1$ for untreated individuals	(7; 8; 9; 10)
ρ_4	$\rho_4 = 1/1.316$ yr^{-1}	Disease related mortality for untreated individuals	
γ_k, $k = 1, 2, 3$	$\gamma_1 = 1/8.21$ yr^{-1}<br>$\gamma_2 = 1/54.0$ yr^{-1}<br>$\gamma_3 = 1/2.463$ yr^{-1}	Rate of transition from stage k to stage $k+1$ for treated individuals	(7; 9; 11)
γ_4	$\gamma_4 = 1/2.737$ yr^{-1}	Disease related mortality for treated individuals	
h_k, $k = 1, 2, 3, 4$	$h_1 = 2.76$<br>$h_2 = 0.106$<br>$h_3 = 0.642$<br>$h_4 = 0.0$	Infectivity of untreated individuals in stage k of infection	(7; 10)
ϵ	0.01	Infectivity of treated individuals	(7; 12)
q_l, $l = 1, \ldots, 6$	$q_1 = 0.451$<br>$q_2 = 0.353$<br>$q_3 = 0.125$<br>$q_4 = 0.06$<br>$q_5 = 0.01$<br>$q_6 = 0.001$	Initial population fractions in the 6 risk groups	(4; 5; 2)
c	2.54 yr^{-1}	Mean partner change rate	Estimated from WPF sexual behavior data

Notation	Baseline value, unit	Description	Source
c_l, $l = 1, \ldots, 6$	see S2 Table	Partner change rates in the 6 risk groups (yr^{-1})	Calculated from the Weibull distribution
σ^2	see S2 Table	Variance of the partner change rate (yr^{-2})	
λ	5%	Transmission probability per partnership	Corresponds to a plausible range of R_0 for MSM in Western countries (7)
τ^*	$[0\%, 100\%]$	Annual treatment percentage for homogeneous uptake	—
τ_l^*, $l = 1, \ldots, 6$	$[0\%, 100\%]$	Annual treatment percentage in group l for heterogenenous uptake	—
ϕ^*	5%	Annual dropout percentage	—
τ	$-\ln[1 - \tau^*/100\%]$ yr^{-1}	Annual treatment uptake rate for homogeneous uptake	—
τ_l, $l = 1, \ldots, 6$	$-\ln[1 - \tau_l^*/100\%]$ yr^{-1}	Annual treatment uptake rate by group l for heterogenenous uptake	—
ϕ	$-\ln[1 - 0.05]$ yr^{-1}	Annual dropout rate	—
ω	$[0, 1]$	Mixing parameter: fully assortative mixing ($\omega = 0$), fully proportionate mixing ($\omega = 1$)	—

Table 1: Description of the parameters of the model and their baseline values.

References

[1] Rutgers Nisso Group, *Sexual health in the Netherlands.* Delft: Eburon, 2006.

[2] M. Xiridou, R. van Houdt, S. Hahné, R. Coutinho, J. van Steenbergen, and M. Kretzschmar, "Hepatitis B vaccination of men who have sex with men in the Netherlands: should we vaccinate more men, younger men or high-risk men?," *Sexually Transmitted Infections*, vol. 89, pp. 666–671, 2013.

[3] A. Johnson, J. Wadsworth, K. Wellings, and J. Field, *Sexual attitudes and lifestyles.* Oxford: Blackwell Scientific Publications, 1994.

[4] J. R. Williams, D. J. Nokes, G. F. Medley, and R. M. Anderson, "The transmission dynamics of hepatitis B in the UK: a mathematical model for evaluating costs

and effectiveness of immunization programmes," *Epidemiology & Infection*, vol. 116, pp. 71–89, 1996.

[5] R. M. Anderson, G. F. Medley, and D. J. Nokes, "Preliminary analyses of the predicted impacts of various vaccination strategies on the transmission of the hepatitis B virus," in *Proceedings of the conference on the control of hepatitis B: the role of prevention in adolescence*, (London), pp. 95–130, 1991.

[6] R. M. Anderson and R. M. May, *Infectious diseases of humans.* Oxford: Oxford University Press, 1991.

[7] M. E. Kretzschmar, M. F. Schim van der Loeff, P. J. Birrell, D. De Angelis, and R. A. Coutinho, "Prospects of elimination of HIV with test-and-treat strategy," *Proceedings of the National Academy of Sciences USA*, vol. 110, p. 1553815543, 2013.

[8] Collaborative Group on AIDS Incubation and HIV Survival including the CASCADE EU Concerted Action. Concerted Action on SeroConversion to AIDS and Death in Europe. "Time from HIV-1 seroconversion to AIDS and death before widespread use of highly-active antiretroviral therapy: a collaborative re-analysis," *Lancet*, vol. 355, pp. 1131–1137, 2000.

[9] P. J. Birrell, A. M. Presanis, D. De Angelis, and The CASCADE Collaboration, "Multi-state models of HIV progression in homosexual men: an application to the CASCADE collaboration," tech. rep., MRC Biostatistics Unit, 2012.

[10] T. D. Hollingsworth, R. M. Anderson, and C. Fraser, "HIV-1 transmission, by stage of infection," *Journal of Infectious Diseases*, vol. 198, pp. 687–693, 2008.

[11] The CASCADE Collaboration. Concerted Action on SeroConversion to AIDS and Death in Europe. "Survival after introduction of HAART in people with known duration of HIV-1 infection," *Lancet*, vol. 355, pp. 1158–1159, 2000.

[12] R. M. Granich, C. F. Gilks, C. Dye, K. M. De Cock, and B. G. Williams, "Universal voluntary HIV testing with immediate antiretroviral therapy as a strategy for elimination of HIV transmission: a mathematical model," *Lancet*, vol. 373, pp. 48–57, 2009.
