# Supplementary material for: Impact of Heterogeneity in Sexual Behavior on Effectiveness in Reducing HIV Transmission with Test-and-Treat Strategy
Source: PLoS Comput Biol. 2016 Aug 1;12(8):e1005012. doi: 10.1371/journal.pcbi.1005012 (PMC4968843; doi:10.1371/journal.pcbi.1005012)
Supplement: S2 Table — (PDF) [file pcbi.1005012.s011.pdf]

σ^2	α	β	c_1	c_2	c_3	c_4	c_5	c_6
63.4878	0.4	0.763345	0.0509471	0.9408	4.7779	15.7860	49.8954	138.4150
32.6387	0.497474	1.25658	0.127469	1.4348	5.4394	14.1953	35.9707	81.5467
6.43567	1	2.53686	0.685056	2.6073	5.2079	8.3798	13.3694	20.0609
1.75848	2	2.86254	1.38842	2.8733	4.0916	5.1884	6.5610	8.0351

Table 2: The variance, the shape and the scale and shape parameters of the distributions used in the analysis and the respective partner change rates in the 6 risk groups.
